# Conchological and molecular analysis of the “non-scaly” Bornean *Georissa* with descriptions of three new species (Gastropoda, Neritimorpha, Hydrocenidae)

**DOI:** 10.3897/zookeys.840.33326

**Published:** 2019-04-17

**Authors:** Mohd Zacaery Khalik, Kasper P. Hendriks, Jaap J. Vermeulen, Menno Schilthuizen

**Affiliations:** 1 Naturalis Biodiversity Center, Vondellaan 55, 2332 AA Leiden, The Netherlands Universiti Malaysia Sarawak Kota Samarahan Malaysia; 2 Institute of Biology Leiden, Faculty of Science, Leiden University, 2333 BE Leiden, The Netherlands Naturalis Biodiversity Center Leiden Netherlands; 3 Faculty of Resource Science and Technology, Universiti Malaysia Sarawak, 94300 Kota Samarahan, Sarawak, Malaysia Leiden University Leiden Netherlands; 4 Groningen Institute for Evolutionary Life Sciences, Faculty of Mathematics and Natural Sciences, University of Groningen, 9747 AG Groningen, The Netherlands Groningen Institute for Evolutionary Life Sciences, Faculty of Mathematics and Natural Sciences, University of Groningen Groningen Netherlands; 5 JK Art and Science, Lauwerbes 8, 2318 AT Leiden, The Netherlands JK Art and Science Leiden Netherlands; 6 Institute for Tropical Biology and Conservation, Universiti Malaysia Sabah, Jalan UMS, 88400 Kota Kinabalu, Sabah, Malaysia Institute for Tropical Biology and Conservation, Universiti Malaysia Sabah Kota Kinabalu Malaysia

**Keywords:** Gastropods, limestone, morphology, phylogenetic, species delimitation, Sabah, Sarawak Malaysian Borneo

## Abstract

The Bornean representatives of the genus *Georissa* (Hydrocenidae) have small, dextral, conical, calcareous shells consisting of ca. three teleoconch whorls. Our recent study on the *Georissa* of Malaysian Borneo has revealed high intra- and inter-specific variation in the “scaly” group (a group of species with striking scale-like surface sculpture). The present study on the “non-scaly” *Georissa* is the continuation of the species revision for the genus. The “non-scaly” species are also diverse in shell sculptures. This informal group comprises *Georissa* with subtle spiral and/or radial sculpture. The combination of detailed conchological assessment and molecular analyses provides clear distinctions for each of the species. Conchological, molecular, and biogeographic details are presented for 16 species of “non-scaly” *Georissa*. Three of these are new to science, namely *Georissacorrugata***sp. n.**, *Georissainsulae***sp. n.**, and *Georissatrusmadi***sp. n.**

## Introduction

The genus *Georissa* Blanford, 1864 (Hydrocenidae) comprises minute terrestrial snails, generally characterized by a small, dextral, conical, calcareous shell with ca. three teleoconch whorls (Bandel 2008; Thompson and Dance 1983; Vermeulen et al. 2015). *Georissa* is primarily restricted to environments rich in calcium carbonate (CaCO_3_). They are found in variable abundances on wet and shaded limestone walls or rocks, but occasionally on sandstone rocks and in vegetation that is not associated with a rocky substrate (Haase and Schilthuizen 2007; Khalik et al. 2018). They have a calcareous operculum, constructed in a concentric paucispiral manner and a peg attached at the inner surface. The hemi-spherically shaped protoconch has a distinct microsculpture, which often shows species-specific distinctness (Khalik et al. 2018).

Until recently, simple conchological analyses have been the main approach to describe and study the species of Bornean *Georissa*. In our recent systematic study of the “scaly” *Georissa* (see Khalik et al. 2018), however, we combined molecular, detailed conchological examination and biogeographic data of each species to assist in the process of species delimitation. We have revealed that this group of minute land snails has high intra- and inter-specific variation, especially in shell shape, size, aperture, and sculptural characters, as well as high allopatric diversity. Here, we provide a complete list of known “scaly” *Georissa* of Borneo: *G.scalinella* (van Benthem-Jutting, 1966); *G.saulae* (van Benthem-Jutting, 1966); *G.hosei* Godwin-Austen, 1889; *G.anyiensis* Khalik et al., 2018; *G.muluensis* Khalik et al., 2018; *G.hadra* Thompson & Dance, 1983; *G.kobelti* Gredler, 1902; *G.niahensis* Godwin-Austen, 1889; *G.silaburensis* Khalik et al., 2018; *G.bauensis* Khalik et al., 2018; *G.pyrrhoderma* Thompson & Dance, 1983; *G.kinabatanganensis* Khalik et al., 2018; *G.sepulutensis* Khalik et al., 2018. Striking allopatric patterns are well-known from several other microsnail taxa of Southeast Asia (see Liew et al. 2014, Hoekstra and Schilthuizen 2011, Rundell 2008, Tongkerd et al. 2004). These studies have led to the realisation that the geographic variation of different populations needs to be well understood and used as an important guideline for species delimitation. Previous phylogenetic studies on the Bornean *Georissa* based on 16S and CO1 mtDNA allowed species to be recognised as monophyletic clades. There were at least two exceptions to this pattern, *G.kobelti* and *G.saulae*, which are paraphyletic with respect to the locally endemic, conchologically distinct *G.niahensis* and *G.filiasaulae*, respectively (Khalik et al. 2018; Schilthuizen et al. 2005). Such paraphyletic patterns are not unexpected when microgeographic speciation yields recently evolved; locally endemic species branched off from more widespread ancestors (Schilthuizen and Gittenberger 1996).

In this paper, the second part of our work on the Bornean *Georissa*, we apply the same approach of combining information from multiple datasets to 16 species of Bornean *Georissa* that belong to the informal “non-scaly” group, characterised mainly by weak to strong spiral and/or radial sculptures without conspicuous scale-like sculpture on the shell. We also present the phylogenetic relationships among all Bornean *Georissa* and their distribution. We describe three species new to science, namely *Georissacorrugata* sp. n., *Georissainsulae* sp. n., and *Georissatrusmadi* sp. n.

## Materials and methods

### Fieldwork and collection material

We examined collection material from:

**RMNH**Naturalis Biodiversity Center (previously collection from Rijksmuseum van Natuurlijke Historie), Leiden,

**ZMA**Naturalis Biodiversity Center (previously collection from Zoological Museum of Amsterdam), Leiden,

**NHMUK**Natural History Museum, London,

**BORN**Borneensis Collection, Universiti Malaysia Sabah,

**MZU**Zoology Museum, Universiti Malaysia Sarawak,

**MFN**Museum für Naturkende, Berlin, and,

**JJV** Jaap Vermeulen (personal collection).

We conducted series of fieldwork at limestone outcrops in Malaysian Borneo between September 2015 and October 2018. We searched for living *Georissa* on the limestone walls and rocks, loose organic matter, and living leaves. The collected specimens were directly sorted and stored in sample tubes with ~96% ethanol. Ca. 5 liters of soil and leaf litter were sampled at every sampling location, which were later sieved and soaked in water to collect the empty shells by flotation (Vermeulen and Whitten 1998). The floating organic matter was extracted and dried. The shells of *Georissa* were manually picked from the dried organic matter using the stereomicroscope and sorted. The holotypes, paratypes, and other materials were deposited at MZU, BORN, and RMNH.

### Morphological analysis

**Microscopy.** The shells were observed under a stereomicroscope for species identification and detailed examination at 40–100 × magnification. The 2-dimensional images of the individual shell of each *Georissa* species were captured in three views, namely apertural (aperture as the frontal view), side (the right side of the shell as the frontal view), and rear (umbilical region as the frontal view) views, using AxioCamMRc5, Zeiss PlanApo S 1.0 × FWD 60.0mm lenses. The apertural view images of each individual shell were then measured to obtain shell height (SH), shell width (SW), aperture height (AH), and aperture width (AW).

**Scanning electron microscopy (SEM).** We selected a representative adult shell of each species for detailed character examination by using scanning electron microscopy. The shells were first cleaned in sodium hypochloride, dried and then sputter-coated with Pd/Pt coating agent. We used a JEOL JSM-6480LV machine for SEM imaging to obtain detailed shell characters of the teleoconch and protoconch.

**Micro-computed tomography (µ-CT).** The µ-CT scanning was carried out using an Xradia 520 Versa X-ray Microscope (see Suppl. material 1 for µ-CT scanning parameters). We obtained ca. 995 layers of X-ray images of an individual shell per scanning, which we then used to reconstruct a composite 3-dimensional image of the shell. These images were then segmented in Avizo ver. 9.4.0 (FEI Company), to examine the operculum, peg, and inner part of the shell.

### Molecular analysis

**DNA extraction**. The sample preparation prior to DNA extraction procedure followed the method from Khalik et al. (2018). We extracted the genomic DNA from 52 individuals using the Qiagen DNeasy Blood and Tissue kit, and applied the protocol provided by the manufacturer.

**DNA amplification**. We used the primer pairs LR-J-12887 5’-CCGGTCTGAACTCAGATCACGT-3’ (forward) and LR-N-13398 5’-CGCCTGTTTAACAAAAAACAT-3’ (reverse) (Schilthuizen et al. 2005) to amplify a fragment of 458–466 bp of 16S gene, and LCO1490 5’-GGTCAACAAATCATAAAGATATTGG-3’ (forward) and HCO2198 5’-TAAACTTCAGGGTGACCAAAAAATCA-3’ (reverse) (Folmer et al. 1994) to amplify a fragment of 585–603 bp CO1 gene. We amplified both these mtDNA regions on a BIO-RAD C1000 Touch Thermal Cycler. The PCR master mix and amplification procedures followed Khalik et al. (2018).

**DNA Sequencing**. PCR products were sent to BaseClear B.V. (Leiden, The Netherlands) and Sanger sequenced in forward and reverse directions using the ABI3730XL sequencer, Life Technologies.

### Sequence alignment and phylogenetic analyses

**Sequence data**. From GenBank, we downloaded 16S and CO1 mtDNA sequences of representatives of the “scaly” group species, *G.gomantonensis* (Khalik et al. 2018), a full mitochondrial genome of *G.similis* (Uribe et al. 2016), and, as an outgroup, *Bathyneritanaticoidea* (Arellano et al. 2016). We extracted the 16S and CO1 regions from the *G.similis* full mitochondrial genome to be included among the sequences in our phylogenetic analysis. The newly sequenced data were assembled using *de novo* Geneious 10.2.3 assembler, manually edited, and trimmed for ambiguities. This resulted in a total of 68 and 55 sequences of 16S and CO1 mtDNA, respectively. Sequences were deposited in GenBank via BankIt (https://www.ncbi.nlm.nih.gov/WebSub/) and BOLD (http://boldsystems.org/).

**Sequence alignment.** The 16S and CO1 mtDNA sequences were aligned to their respective genes using default parameters of MUSCLE (Edgar 2004). The alignments were manually checked and edited.

**Phylogenetic inference**. The alignment of CO1 mtDNA was set to invertebrate mitochondrial genetic code at the third reading frame. The best fit nucleotide substitution models of the concatenated 16S and CO1 sequence alignment was determined using ModelFinder (Kalyaanamoorthy et al. 2017) based on corrected Akaike Information Criterion (AICc). The best-fit nucleotide model for the concatenated sequence alignment is GTR+F+R4.

**Phylogenetic analysis**. We performed a maximum likelihood analysis using the concatenated alignment using GTR+F+R4 nucleotide substitution model with ultrafast bootstrapping (5000 replicates) (Hoang et al. 2017) in IQ-TREE 1.6.3 (Nguyen et al. 2015). We used MrBayes 3.2.6 (Huelsenbeck and Ronquist 2001) for Bayesian Inference using the following settings: GTR+I+G nucleotide substitution model; 1,100,000 number of generations; tree subsampling for every 200 generation; 100,000 burn-in length; 4 heated chains with heated chain temperature at 0.2. Details of the newly sequenced data and their accession number are listed in Table 1.

**Table 1.** List of specimens used in molecular analyses.

No.	Species	Voucher No.	Species name_sequence origin_location Town/District/Division, State. GPS coordinate	GenBank Accession No.	
				16S	CO1
1	*Georissasaulae* (van Benthem Jutting, 1966)	BOR/MOL 2663-2667	G.saulae_AY547385_Sinobang	AY547385 (Schilthuizen et al. 2012)	n/a
			Batu Sinobang, Sabah.		
			04°48.04'N, 116°37.03'E		
2	*Georissasaulae* (van Benthem Jutting, 1966)	BOR/MOL 12770	G.saulae_Sau-001_Pungiton	MG982262 (Khalik et al. 2018)	MK722149
			Sepulut Valley, Gua Pungiton, Sabah.		
			04°42.41'N, 116°36.04'E		
3	*Georissasaulae* (van Benthem Jutting, 1966)	BOR/MOL 12770	G.saulae_Sau-002_Pungiton	MG982263 (Khalik et al. 2018)	MK722150
			Sepulut Valley, Gua Pungiton, Sabah.		
			04°42.41'N, 116°36.04'E		
4	*Georissafiliasaulae* Haase & Schilthuizen, 2007	BOR/MOL 12768	G.filiasaulae_002_Pungiton	MK411785	MK505425
			Sepulut Valley, Gua Pungiton, Sabah.		
			04°42.41'N, 116°36.04'E		
5	*Georissafiliasaulae* Haase & Schilthuizen, 2007	BOR/MOL 12768	G.filiasaulae_003_Pungiton	MK411786	MK505426
			Sepulut Valley, Gua Pungiton, Sabah.		
			04°42.41'N, 116°36.04'E		
6	*Georissafiliasaulae* Haase & Schilthuizen, 2007	BOR/MOL 12768	G.filiasaulae_005_Pungiton	MK411787	MK505427
			Sepulut Valley, Gua Pungiton, Sabah.		
			04°42.41'N, 116°36.04'E		
7	*Georissapachysoma* Vermeulen & Junau, 2007	MZU/MOL 17.63	G.pachysoma_BSM2-01_Bukit Sarang	MK411789	MK505443
			Bukit Sarang, Bintulu, Sarawak.		
			02°39.31'N, 113°02.47'E		
8	*Georissapachysoma* Vermeulen & Junau, 2007	MZU/MOL 17.63	G.pachysoma_BSM2-02_Bukit Sarang	MK411788	MK505442
			Bukit Sarang, Bintulu, Sarawak.		
			02°39.31'N, 113°02.47'E		
9	*Georissapachysoma* Vermeulen & Junau, 2007	MZU/MOL 17.63	G.pachysoma_BSM2-03_Bukit Sarang	MK411791	MK505441
			Bukit Sarang, Bintulu, Sarawak.		
			02°39.31'N, 113°02.47'E		
10	*Georissapachysoma* Vermeulen & Junau, 2007	MZU/MOL 17.63	G.pachysoma_BSM2-04_Bukit Sarang	MK411790	MK505440
			Bukit Sarang, Bintulu, Sarawak.		
			02°39.31'N, 113°02.47'E		
11	*Georissasimilis* Smith, 1893	MZU/MOL 16.14	G.similis_E001_Batu Batangan	MK411792	MK505446
			Batu Batangan, Sabah.		
			05°27.61'N, 118°06.17'E		
12	*Georissasimilis* Smith, 1893	MZU/MOL 16.14	G.similis_E002_Batu Batangan	MK411795	MK505444
			Batu Batangan, Sabah.		
			05°27.61'N, 118°06.17'E		
13	*Georissasimilis* Smith, 1893	MZU/MOL 16.14	G.similis_E003_Batu Batangan	MK411793	n/a
			Batu Batangan, Sabah.		
			05°27.61'N, 118°06.17'E		
14	*Georissasimilis* Smith, 1893	MZU/MOL 16.14	G.similis_E004_Batu Batangan	MK411794	MK505445
			Batu Batangan, Sabah.		
			05°27.61'N, 118°06.17'E		
15	*Georissabangueyensis* Smith, 1895	RMNH/MOL 5005090	G.bangueyensis_KPH01627.01_NewLocation1	MK403002	MH254770
			New Location 1, Kinabatangan River, Sabah		
			05°27.40'N, 118°08.76'E		
16	*Georissabangueyensis* Smith, 1895	RMNH/MOL 5005090	G.bangueyensis_KPH01627.02_NewLocation1	MK402999	MH254645
			New Location 1, Kinabatangan River, Sabah		
			05°27.40'N, 118°08.76'E		
17	*Georissabangueyensis* Smith, 1895	RMNH/MOL 5005052	G.bangueyensis_KPH01589.01_NewLocation1	MK402996	n/a
			New Location 1, Kinabatangan River, Sabah		
			05°27.40'N, 118°08.76'E		
18	*Georissabangueyensis* Smith, 1895	RMNH/MOL 5005052	G.bangueyensis_KPH01589.02_NewLocation1	MK402993	MH254230
			New Location 1, Kinabatangan River, Sabah		
			05°27.40'N, 118°08.76'E		
19	*Georissabangueyensis* Smith, 1895	RMNH/MOL 5005052	G.bangueyensis_KPH01589.05_NewLocation1	MK402998	MH254559
			New Location 1, Kinabatangan River, Sabah		
			05°27.40'N, 118°08.76'E		
20	*Georissabangueyensis* Smith, 1895	RMNH/MOL 5005057	G.bangueyensis_KPH01594.01_NewLocation1	MK402997	MH254416
			New Location 1, Kinabatangan River, Sabah		
			05°27.40'N, 118°08.76'E		
21	*Georissaflavescens* Smith, 1895	BOR/MOL 7660	G.flavescens_KPH02157.12_Pangi	MK402995	MH254340
			Batu Pangi, Kinabatangan valley, Sabah.		
			05°32.01'N, 118°18.24'E		
22	*Georissaflavescens* Smith, 1895	BOR/MOL 7638	G.flavescens_KPH02135.11_Pangi	MK402989	MH254024
			Batu Pangi, Kinabatangan valley, Sabah.		
			05°31.89'N, 118°18.37'E		
23	*Georissaflavescens* Smith, 1895	BOR/MOL 7626	G.flavescens_KPH02123.07_Tomanggong Besar	MK403001	MH254706
			Batu Tomanggong Besar, Kinabatangan valley, Sabah.		
			05°31.83'N, 118°18.26'E		
24	*Georissaflavescens* Smith, 1895	BOR/MOL 7293	G.flavescens_KPH01725.08_Tomanggong Besar	MK402992	MH254160
			Batu Tomanggong Besar, Kinabatangan valley, Sabah.		
			05°31.52'N, 118°18.41'E		
25	*Georissaflavescens* Smith, 1895	BOR/MOL 7638	G.flavescens_KPH02135.08_Pangi	MK402990	MH254028
			Batu Pangi, Kinabatangan valley, Sabah.		
			05°31.89'N, 118°18.37'E		
26	*Georissaflavescens* Smith, 1895	BOR/MOL 7416	G.flavescens_KPH01860.09_Tomanggong Besar	MK403003	MH254769
			Batu Tomanggong Besar, Kinabatangan valley, Sabah.		
			05°31.38'N, 118°17.89'E		
27	*Georissaflavescens* Smith, 1895	BOR/MOL 7299	G.flavescens_KPH01733.03_Tomanggong Besar	MK402994	MH254313
			Batu Tomanggong Besar, Kinabatangan valley, Sabah.		
			05°31.33'N, 118°18.06'E		
28	*Georissaflavescens* Smith, 1895	BOR/MOL 7294	G.flavescens_KPH01727.13_Tomanggong Besar	n/a	MH254614
			Batu Tomanggong Besar, Kinabatangan valley, Sabah.		
			05°31.46'N, 118°18.14'E		
29	*Georissanephrostoma* Vermeulen et al., 2015	MZU/MOL 17.29	G.nephrostoma_K001_Keruak	MK411797	MK505439
			Batu Keruak, Kinabatangan valley, Sabah.		
			05°32.291'N, 118°18.376'E		
30	*Georissanephrostoma* Vermeulen et al., 2015	MZU/MOL 17.29	G.nephrostoma_K002_Keruak	MK411798	n/a
			Batu Keruak, Kinabatangan valley, Sabah.		
			05°32.291'N, 118°18.376'E		
31	*Georissanephrostoma* Vermeulen et al., 2015	MZU/MOL 17.29	G.nephrostoma_K003_Keruak	MK411800	n/a
			Batu Keruak, Kinabatangan valley, Sabah.		
			05°32.291'N, 118°18.376'E		
32	*Georissanephrostoma* Vermeulen et al., 2015	MZU/MOL 17.29	G.nephrostoma_K004_Keruak	MK411796	n/a
			Batu Keruak, Kinabatangan valley, Sabah.		
			05°32.291'N, 118°18.376'E		
33	*Georissanephrostoma* Vermeulen et al., 2015	MZU/MOL 17.29	G.nephrostoma_K005_Keruak	MK411799	n/a
			Batu Keruak, Kinabatangan valley, Sabah.		
			05°32.291'N, 118°18.376'E		
34	*Georissaxesta* Thompson & Dance, 1983	BOR/MOL 7258	G.xesta_KPH02048.12_Materis	MK403000	MH254698
			Materis, Kinabatangan valley, Sabah.		
			05°31.39'N, 118°10'E		
35	*Georissaxesta* Thompson & Dance, 1983	BOR/MOL 7303	G.xesta_KPH01738.05_Ulu Resang	MK402991	MH254122
			Ulu Sungai Resang, Kinabatangan valley, Sabah.		
			05°30.67'N, 118°20.39'E		
36	*Georissaxesta* Thompson & Dance, 1983	BOR/MOL 7311	G.xesta_KPH01746.06_Ulu Resang	n/a	MH254082
			Ulu Sungai Resang, Kinabatangan valley, Sabah.		
			05°31.16'N, 118°19.78'E		
37	*Georissahungerfordi* Godwin-Austen, 1889	MZU/MOL 16.11	G.hungerfordi_G001_Mawah	MK411771	n/a
			Gunong Mawah, Padawan/Penrissen, Sarawak.		
			01°16.15'N, 110°15.46'E		
38	*Georissahungerfordi* Godwin-Austen, 1889	MZU/MOL 16.11	G.hungerfordi_G002_Mawah	MK411773	n/a
			Gunong Mawah, Padawan/Penrissen, Sarawak.		
			01°16.15'N, 110°15.46'E		
39	*Georissahungerfordi* Godwin-Austen, 1889	MZU/MOL 16.11	G.hungerfordi_G003_Mawah	MK411770	MK505432
			Gunong Mawah, Padawan/Penrissen, Sarawak.		
			01°16.15'N, 110°15.46'E		
40	*Georissahungerfordi* Godwin-Austen, 1889	MZU/MOL 16.11	G.hungerfordi_G004_Mawah	MK411772	n/a
			Gunong Mawah, Padawan/Penrissen, Sarawak.		
			01°16.15'N, 110°15.46'E		
41	*Georissahungerfordi* Godwin-Austen, 1889	MZU/MOL 16.10	G.hungerfordi_I001_Regu	MK411775	MK505428
			Regu, Padawan/Penrissen, Sarawak.		
			01°12.82'N, 110°16.82'E		
42	*Georissahungerfordi* Godwin-Austen, 1889	MZU/MOL 16.10	G.hungerfordi_I002_Regu	MK411774	MK505438
			Regu, Padawan/Penrissen, Sarawak.		
			01°12.82'N, 110°16.82'E		
43	*Georissahungerfordi* Godwin-Austen, 1889	MZU/MOL 16.10	G.hungerfordi_I003_Regu	MK411777	MK505437
			Regu, Padawan/Penrissen, Sarawak.		
			01°12.82'N, 110°16.82'E		
44	*Georissahungerfordi* Godwin-Austen, 1889	MZU/MOL 16.10	G.hungerfordi_I004_Regu	MK411776	MK505436
			Regu, Padawan/Penrissen, Sarawak.		
			01°12.82'N, 110°16.82'E		
45	*Georissahungerfordi* Godwin-Austen, 1889	MZU/MOL 16.13	G.hungerfordi_H001_Sirat	MK411784	MK505431
			Gunong Sirat, Padawan/Penrissen, Sarawak.		
			01°12.42'N, 110°16.52'E		
46	*Georissahungerfordi* Godwin-Austen, 1889	MZU/MOL 16.13	G.hungerfordi_H002_Sirat	MK411783	MK505430
			Gunong Sirat, Padawan/Penrissen, Sarawak.		
			01°12.42'N, 110°16.52'E		
47	*Georissahungerfordi* Godwin-Austen, 1889	MZU/MOL 16.13	G.hungerfordi_H003_Sirat	MK411778	MK505429
			Gunong Sirat, Padawan/Penrissen, Sarawak.		
			01°12.42'N, 110°16.52'E		
48	*Georissahungerfordi* Godwin-Austen, 1889	MZU/MOL 16.13	G.hungerfordi_H004_Sirat	MK411782	n/a
			Gunong Sirat, Padawan/Penrissen, Sarawak.		
			01°12.42'N, 110°16.52'E		
49	*Georissahungerfordi* Godwin-Austen, 1889	MZU/MOL 16.12	G.hungerfordi_F001_Duai	MK411780	MK505435
			Gunong Seduai, Padawan/Penrissen, Sarawak.		
			01°12.25'N, 110°17.00'E		
50	*Georissahungerfordi* Godwin-Austen, 1889	MZU/MOL 16.12	G.hungerfordi_F002_Duai	MK411781	MK505434
			Gunong Seduai, Padawan/Penrissen, Sarawak.		
			01°12.25'N, 110°17.00'E		
51	*Georissahungerfordi* Godwin-Austen, 1889	MZU/MOL 16.12	G.hungerfordi_F004_Duai	MK411779	MK505433
			Gunong Seduai, Padawan/Penrissen, Sarawak.		
			01°12.25'N, 110°17.00'E		
52	*Georissainsulae* sp. n.	MZU/MOL 18.02	G.insulae_Man_001	MK411801	n/a
			Pulau Mantanani Besar, Sabah.		
			06°43.06'N, 116°20.50'E		
53	*Georissainsulae* sp. n.	MZU/MOL 18.02	G.insulae_Man_002	MK411803	n/a
			Pulau Mantanani Besar, Sabah.		
			06°43.06'N, 116°20.50'E		
54	*Georissainsulae* sp. n.	MZU/MOL 18.02	G.insulae_Man_003	MK411804	n/a
			Pulau Mantanani Besar, Sabah.		
			06°43.06'N, 116°20.50'E		
55	*Georissainsulae* sp. n.	MZU/MOL 18.02	G.insulae_Man_004	MK411802	n/a
			Pulau Mantanani Besar, Sabah.		
			06°43.06'N, 116°20.50'E		

### Species delimitation and description

Species delimitation of the “non-scaly” group Bornean *Georissa* was carried out based on detailed examination of the shell characters which are exclusive to the group, combined with the molecular analyses. While morphological analysis is widely accepted for species identification in gastropods, this conventional way of species delimitation could become very challenging when applied to the genus *Georissa* which show high morphological variation within and between populations. For this reason, we applied a similar species delimitation approach as done in the “scaly” group *Georissa* (Khalik et al. 2018). In view of the considerations given in Khalik et al. (2018), we refrained from web-based species delimitation in this case.

### CO1 genetic divergence

CO1 genetic divergence was performed to determine the genetic distances between species of the “non-scaly” group *Georissa*. We conducted genetic distance analysis within and between species groups. We computed pairwise genetic distances of CO1 sequence alignment based on the nucleotide substitution model Kimura 2-parameter in MEGA v. 7.0.26 (Kumar et al. 2016) which includes the transition + transversion, gamma distribution, and 1000 bootstraps for variance estimate. We conducted the analysis based on CO1 sequence data of 40 individuals comprised of nine species, including three newly described species.

## Results and discussion

### Morphological and phylogenetic analyses

The “non-scaly” *Georissa* from Borneo are characterised by the simple spiral and/or radial sculpture on the shell, unlike the distinct scale-like structures of the “scaly” group. These two informal groups of *Georissa* could be used as an initial framework for future species identification. Previously, Thompson and Dance (1983) divided the Bornean *Georissa* into four groups, namely the “*hosei*”, “*borneensis*”, “*everetti*”, and “*williamsi*” groups. The “*hosei*” group and a species of the “*borneensis*” group (i.e., *G.pyrrhoderma*) are species with scaly sculpture. Thompson and Dance (1983) included *G.monterosatiana* from Peninsular Malaysia in the “*hosei*” group, which does not have obvious scales on the shell. The rest of the groups of Thompson and Dance (1983) consist of the “non-scaly” species, which were further distinguished based on their colour and ribbing. Although shell colour may help in species-level taxonomy, we suggest not to use colour as a character for species grouping, given the wide range of shell colour variation in most Bornean *Georissa*.

Our previous work on the “scaly” group Bornean *Georissa* (Khalik et al. 2018) together with this present study on the “non-scaly” group have resulted in a complete revision of the Bornean *Georissa*. To date, we recognise 29 species of Bornean *Georissa*, of which 13 are in the “scaly” group and 16 are in the “non-scaly” group. Since we have studied and examined all shell materials from BORN, MZU, ZMA, RMNH, MFN, NHMUK, and JJV, we find that it is useful to highlight some issues related to the “non-scaly” group that could be beneficial for future understanding. Firstly, the name *G.williamsi* was mentioned in several publications to refer to a species with distinct spiral ribs (Thompson and Dance 1983; Clements et al. 2008; Nurinsiyah et al. 2016; Maassen 2003; O’Loughlin and Green 2016; Vermeulen and Whitten 1998). After examination of the holotype of *G.williamsi* in the NHMUK, we find that this species name has often been misapplied. The images provided by Thompson and Dance (1983, figs 66–68), Phung et al. (2017, fig. 8C), and Vermeulen and Whitten (1998, fig. 15) show entirely different spiral sculpture than the ‘true’ *G.williamsi*. Based on the taxonomy presented in this paper, the specimens illustrated in Thompson and Dance (1983) are *G.bangueyensis* Smith, 1895; those in Phung et al. (2017) are *G.insulae* sp. n.; and that in Vermeulen and Whitten (1998) is *G.javana* Möllendorff, 1897.

There is a similar confusion with *G.borneensis*, a name widely applied to both *G.similis* and *G.corrugata* sp. n. in the collection materials. Schilthuizen et al. (2003) mentioned *G.similis*, but it is presently not sure if this refers to the true *G.similis* or otherwise, because the collection numbers of the specimens used in their studies (materials deposited in BORN/RMNH) was not mentioned. *Georissasimilis* and *G.corrugata* are conchologically distinct from *G.borneensis* (see detailed description in Systematic part).

Clements et al. (2008) and Schilthuizen et al. (2003, 2011) refer to several species of “non-scaly” *Georissa*, namely, *G.borneensis*, *G.bangueyensis*, *G.similis*, and *G.williamsi*. Again, we cannot be sure whether the specimens were correctly assigned since we could not examine the materials studied by these authors, due to similar case as above.

On the one hand, we find that “non-scaly” *Georissa* have strongly supported monophyletic groups with bootstrap and posterior output values in our phylogenetic analyses, ranging from 96–100 and 100, respectively. This corresponds to conchological characters of the respective taxa. On the other hand, we find *G.xesta* is paraphyletic. Discussions for each species treatment are in the Systematic part.

### CO1 genetic divergence

Species delimitation based solely on morphological analysis of this group of closely related minute gastropods could be challenging, especially when the studied taxa have high intra-specific variation (see Khalik et al. 2018; Liew et al. 2014). The analysis of molecular data provides a large benefit in the process of species delimitation. Previous systematic studies of gastropods have reported to successfully delimit the studied taxa to a species level by using CO1 divergence (see Boeters and Knebelsberger 2012; Liew et al. 2014, Khalik et al. 2018; Puillandre et al. 2012), but provide no specific genetic barriers for each studied taxon.

The CO1 genetic divergence (Table 2) shows the Kimura 2-parameter distances within a group of species and net average distances between groups of *Georissa* sequences. This reveals that between-species genetic divergence of the “non-scaly” species exceeded 0.10, with the exception of *G.xesta* vs. *G.flavescens*, *G.xesta* vs. *G.bangueyensis*, and *G.xesta* vs. *G.nephrostoma*. Although the divergences of these species pairs are considerably low, they comprise groups of species with distinct morphological characters. This is similar to what was found with the “scaly” *Georissa*, for example, *G.silaburensis* vs. *G.bauensis* is a conchologically distinct species pair that has a CO1 divergence as low as 0.04 (Khalik et al. 2018). We also find that the intraspecific divergence within each “non-scaly” species is equal or does not exceed 0.05, with the exception of *G.xesta* (0.11).

Hoekstra and Schilthuizen (2011) suggested that intraspecific divergence for a limestone-dwelling microsnail (*Gyliotrachelahungerfordiana* Möllendorff, 1886) of Peninsular Malaysia would not exceed 0.10, which we find in the Bornean *Georissa* as well, with the exception of *G.xesta*. We find that the genetic divergence analysis of the Bornean *Georissa* provides useful information for species delimitation. There is, however, no specific genetic divergence limit that separates intraspecific from interspecific distances, since the divergence within a species and divergence between species often overlap (the highest value for intraspecific divergence = 0.11, while the lowest value for intraspecific divergence = 0.03).

**Table 2.** Intra- and inter-specific divergence of partial CO1 sequences of nine species of the “non-scaly” *Georissa*.

		Divergence within group	Number of specimens	1	2	3	4	5	6	7	8	9
1	* G. gomantonensis *	0.00	2									
2	* G. filiasaulae *	0.00	3	0.24								
3	* G. hungerfordi *	0.05	11	0.21	0.22							
4	* G. pachysoma *	<0.01	4	0.22	0.25	0.20						
5	* G. similis *	<0.01	3	0.22	0.22	0.20	0.17					
6	* G. flavescens *	0.03	8	0.19	0.22	0.16	0.20	0.19				
7	* G. bangueyensis *	<0.01	5	0.22	0.23	0.17	0.23	0.20	0.11*			
8	* G. nephrostoma *	–	1	0.20	0.23	0.16	0.17	0.17	0.13	0.12		
9	* G. xesta *	0.11	3	0.17	0.18	0.13	0.12	0.13	0.09*	0.07*	0.03*	

*The average number of net base substitutions per site between species is equal or lower than 0.11, which is lower or equal to the highest number of base substitutions per site within a “non-scaly” species.

## Systematic part

### Class Gastropoda Cuvier, 1797

#### Family Hydrocenidae Troschel, 1856

##### Genus *Georissa* Blanford, 1864

###### “Non-scaly” group

We previously described the first informal group of Bornean *Georissa*, the “scaly” group which consists of 13 species (Khalik et al. 2018). In the current paper, we describe the remaining group of Bornean *Georissa*, consisting of 16 species which do not have conspicuous scale sculpture and are characterised mainly based on species-specific patterns of more subtle radial and/or spiral sculpture. Our “non-scaly” group corresponds to Thompson and Dance’s (1983) “*williamsi*”, “*everetti*” and “*borneensis*” (p.p.) groups. A species of the “*borneensis* group”, *Georissapyrrhoderma* Thompson & Dance, 1983 has been previously included by us in the “scaly” group (Khalik et al. 2018).

**General conchological description of a “non-scaly” group representative.***Protoconch*. Colour (in living or freshly dead specimens): white, yellowish green, orange, red, or brown. Sculpture pattern: smooth (no sculpture on the protoconch), straight lines (the sculpture is raised in a pattern of straight lines), rounded to ellipsoidal (the sculpture is rounded and/or ellipsoidal), mixed (a combination of more than one sculpture patterns), or irregular (the present sculpture comprises of no uniform shape or pattern). *Teleoconch*. Colour (in living or freshly dead specimens): white, yellowish green, orange, red, or brown. First and subsequent whorls: convex (the whorls are partially circular in shape), rounded (the whorls are semi-circular in shape), and/or flat. Suture: deeply impressed. Shoulder: narrow or extended. Number of whorls: 2 ¼–3 ½. Shell height (SH): 0.62–2.23 mm. Shell width (SW): 0.60–1.82 mm. Shell index (SI=SH/SW): 0.97–1.51. *Shell sculpture*. Radial sculpture: absent or present; if present then either raised in wavy and/or regular form, with narrow or wide interval. Growth lines: weak or strong, for species without clear formation of radial sculpture. Species with radial sculpture normally do not have clear growth lines since these are covered by the radial sculpture; such species generally have a row of nodules at the shoulder close to and parallel to the suture or away from the suture on the whorls. Spiral sculpture: absent, weak or strong, continuous or discontinuous, frequently the orientation is distorted by the radial sculpture (if present). *Columella*. Smooth and translucent. Umbilicus: open or closed. *Aperture*. Shape: semi-elliptic, ovoid or rounded, with straight, concave or convex parietal side, palatal edge either contiguous with the body whorl or with the parietal side. Aperture height (AH): 0.31–1.07 mm. Aperture width (AW): 0.33–1.09 mm. Aperture index (AI = AH/AW): 0.81–1.02. *Peristome*. Simple, thickened inside, sharp toward the edge of the aperture. *Operculum*. Shape: ovoid to rounded, the inner surface of the operculum has a small crater-like structure next to the peg. Peg: straight or curved. The shell dimensions of the “non-scaly” *Georissa* are summarised in Suppl. material 2.

All species of Bornean *Georissa* have a broadly developed callus that fully covers the umbilicus, except *G.leucococca*, which has this callus incompletely developed. Hence, the umbilical region of this species is partially open. *Georissanephrostoma* is the only known Bornean *Georissa* with a ‘bulb’-like callus covering the umbilical region. This is an inflation of the columella along the parietal wall. As a result, the aperture of *G.nephrostoma* is partly obstructed, unlike any other aperture of the Bornean *Georissa*. Of all the “non-scaly” *Georissa*, the operculum is available, except for *G.corrugata*, *G.williamsi*, and *G.leucococca*.

**Habitat and ecology.** Like the “scaly” group, the members of the “non-scaly” group *Georissa* are usually restricted to limestone areas. They can be found on the limestone walls, rocks located in wet and shaded environments, and occasionally at a low density on dry limestone walls and rocks, in the vegetation away from the limestone (e.g., *G.gomantonensis*), on other, non-limestone rocky substrates (e.g., *G.saulae*), and on limestone walls inside cave systems with partial or no exposure to the sunlight (e.g., *G.silaburensis* and *G.filiasaulae*).

**Distribution.** We provide distribution maps of the “non-scaly” *Georissa* of Malaysian Borneo in Figures 3, 4. The species are divided into two distribution maps to avoid overlapping. There are at least twelve species of the “non-scaly” group in Sabah, two species in Sarawak, and another two species in both Sabah and Sarawak.

**Remark.** For the type material that was not examined during this study, there is a note in each of the species treatment that the type specimen was not seen.

In the following systematic descriptions of the “non-scaly” *Georissa*, the species treatment is arranged partly based on the molecular phylogeny (Fig. 2A, B). We start with the description of six species for which no DNA-data are available, namely (i) *Georissaborneensis* Smith, 1893, (ii) *Georissacorrugata* sp. n., (iii) *Georissaeveretti* Smith, 1895, (iv) *Georissawilliamsi* Godwin-Austen, 1889, (v) *Georissatrusmadi* sp. n., and (vi) *Georissaleucococca* Vermeulen, Liew & Schilthuizen, 2015, followed by the remaining ten species, treated in the order in which they appear in the phylogenetic tree. The numbers of individuals of the newly described species are stated in brackets (if available) right after the collection number. The locality data may contain the following Malay words: Batu = rock; Bukit = hill; Gua = cave; Sungai/Sungei/Sg. = river; Gunung/Gunong = mountain; Pulau = island; Kampung = village.

####### Georissa borneensis

Taxon classificationAnimaliaCycloneritidaHydrocenidae

Smith, 1895

Figures 1A , 5A–K

Georissa borneensis Smith, 1895: 126, plate IV fig. 18; Thompson and Dance 1983: 122, figs 18, 61–62.

######## Type locality.

Gomanton, N.E. Borneo.

######## Type material.

**Figure 1.**
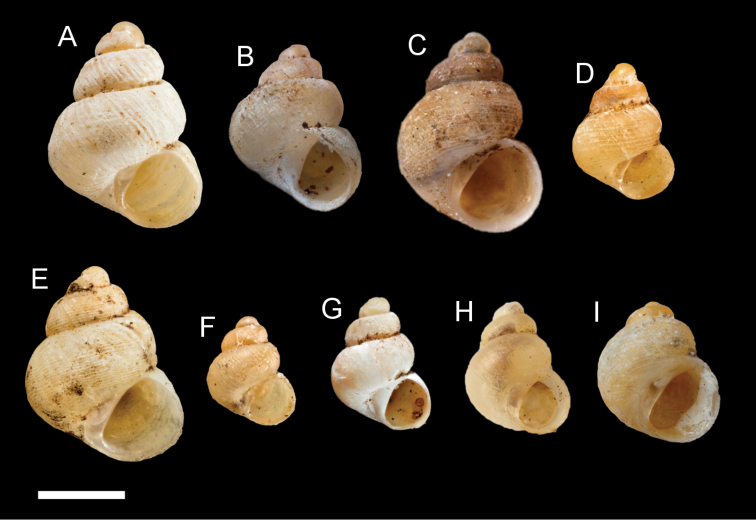
The types specimens of the “non-scaly” *Georissa* of Borneo from NHMUK. **A***Georissaborneensis* Smith, 1895 **B***Georissaeveretti* Smith, 1895 **C***Georissawilliamsi* Godwin-Austen, 1889 **D***Georissahungerfordi* Godwin-Austen, 1889 **E***Georissagomantonensis* Smith, 1893 **F***Georissasimilis* Smith, 1893 **G***Georissaxesta* Thompson & Dance, 1983 **H***Georissabangueyensis* Smith, 1895 **I***Georissaflavescens* Smith, 1895. Scale bar: 1 mm. Photos by NHMUK.

*Lectotype* (Designation by Thompson and Dance 1983) (Fig. 1A). Gomanton, N. Borneo: NHMUK 1894.7.20.61 (glued on paper). *Paralectotypes*. Gomanton, N. Borneo: NHMUK 1894.7.20.62, NHMUK 1894.21.54–57 (glued on paper).

**Figure 2.**
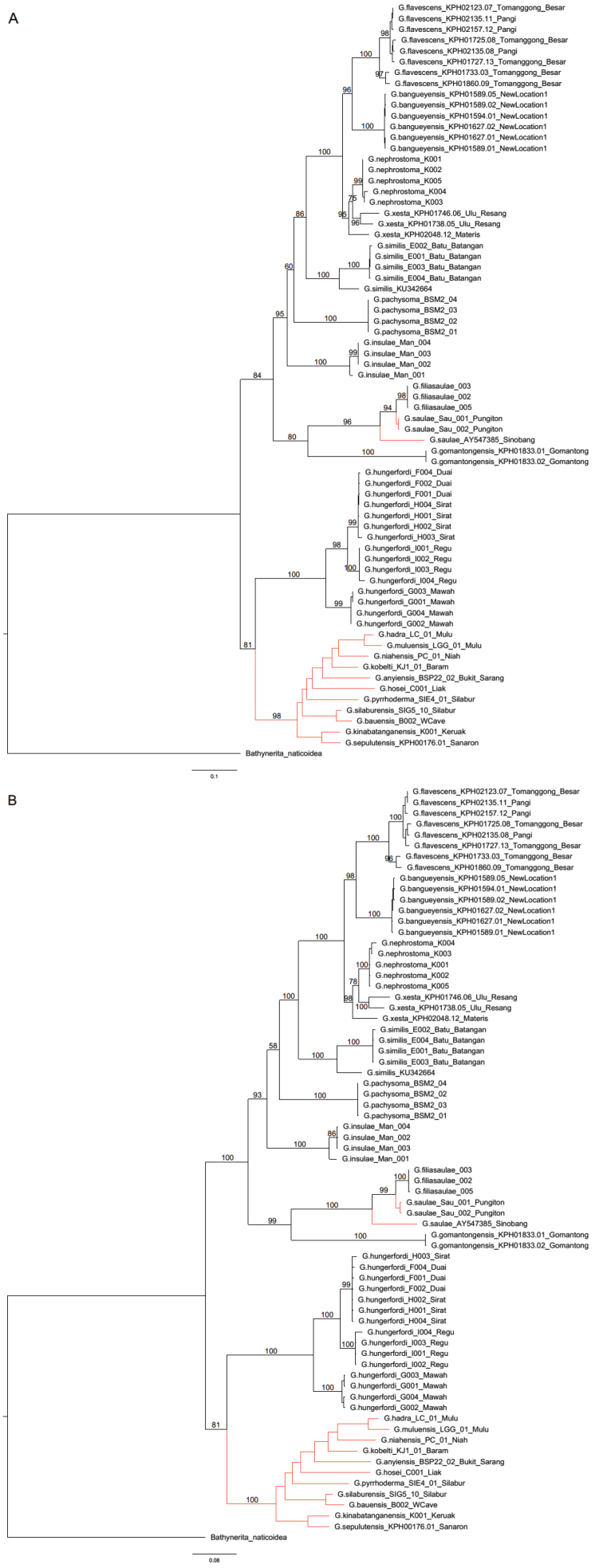
**A** Maximum likelihood phylogenetic reconstruction with ultrafast bootstrapping (5000 replicates) **B** bayesian phylogenetic reconstruction with posterior probabilities, constructed using MrBayes analysis. Phylogenetic analyses were conducted using concatenated sequence alignments of partial 16S and CO1 mtDNA. The analyses consist of 69 ingroup taxa (11 taxa representing the “scaly” group and 58 taxa representing the “non-scaly” group), and *Bathyneritanaticoidea* as an outgroup. “Scaly” taxa in the phylogenies are with the red branches.

**Figure 3.**
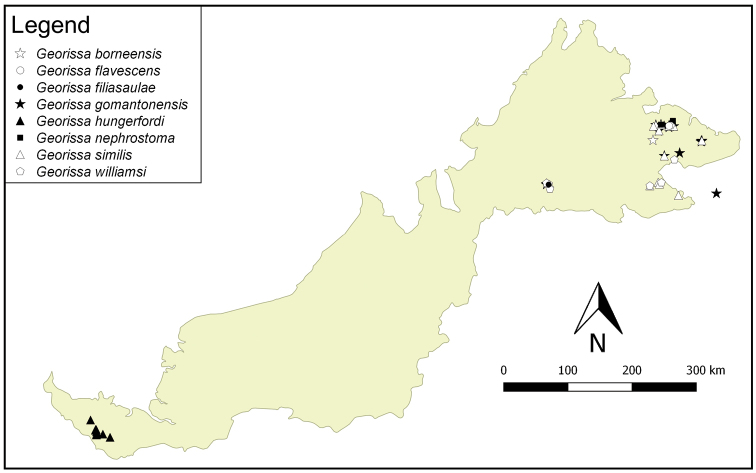
The distribution of eight species of the “non-scaly” *Georissa* of Malaysian Borneo, based on studied materials.

**Figure 4.**
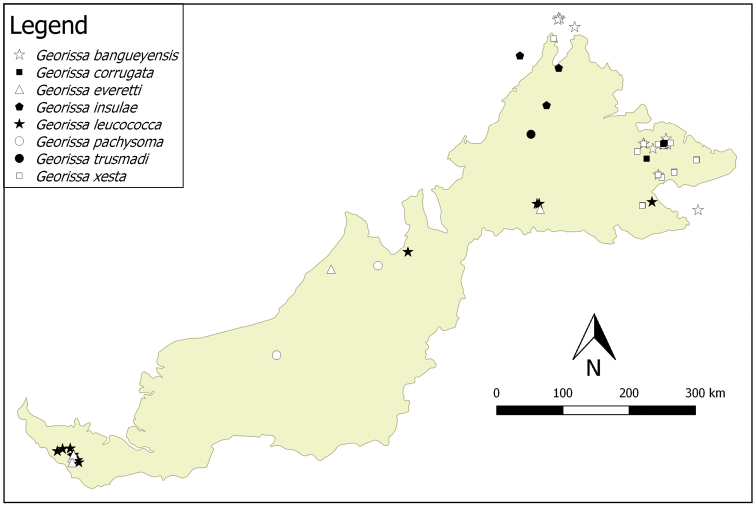
The distribution of another eight species of the “non-scaly” *Georissa* of Malaysian Borneo, based on studied materials.

######## Other material.

**Figure 5.**
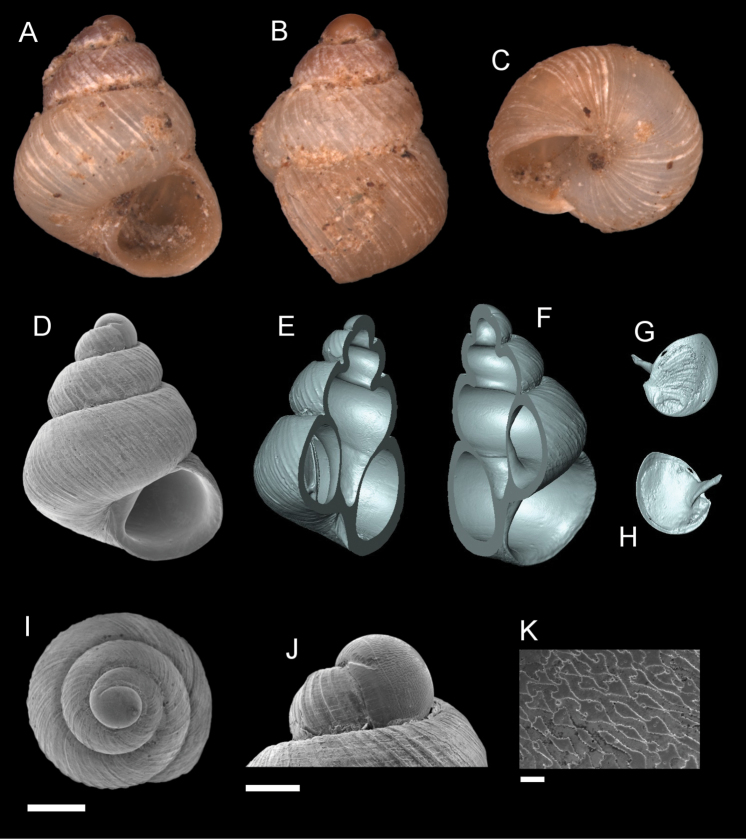
*Georissaborneensis* Smith, 1895. **A–K**ZMA/MOLL 315546 **A, D** shell apertural view **B** shell side view **C** shell rear view **E, F** shell cross-section from 3D model **G, H** operculum frontal and ventral view from 3D model **I** shell top view **J** protoconch side view **K** close up of protoconch from top at 1000 × magnification. Scale bars: 500 µm (**A–I**); 200 µm (**J**); 10 µm (**K**).

N. Borneo: RMNH/MOL 152748, ZMA/MOLL 315546 (Fig. 5). Gomanton, N. Borneo: MFN 47552, MFN 47942. Kinabatangan valley, Gomantong Hill 30 km South of Sandakan, Sandakan Province, Sabah (05°19.20'N, 118°3.60'E): JJV 1613.

######## Description.

*Protoconch*. Colour: white to pale orange, darker than the rest of the shell. Sculpture pattern: irregular sculpture pattern, from base to apex end with no specific sculptural shape. Mesh width: 2.5–8.0 µm. *Teleoconch*. Colour: white to pale orange. First whorl: flat, convex close to the suture. Subsequent whorls: flat, convex and angular at the periphery. Suture: clearly impressed. Shoulder: narrow. Number of whorls: 2 ¾–3 ½. SH: 1.91–2.23 mm. SW: 1.65–1.82 mm. SI: 1.12–1.28. *Shell sculpture*. Radial sculpture: present, weak or flattened, densely sculpted on the whorls, ca. two to three ribs per 0.1 mm. Spiral sculpture: present, but thin and weak, only visible under high magnification (> ×100 magnification), strongest at the first whorl, weaker at subsequent whorls. *Aperture*. Shape: semi-elliptic, straight parietal side, palatal edge contiguous with the body whorl, palatal side tilted and angular, basal side convex. AH: 0.82–1.07 mm. AW: 1.00–1.09 mm. AI: 0.75–1.02.

######## Diagnosis.

The flat whorls that are strongly convex at the periphery, giving the shell an angular shape, are diagnostic. The sculpture of *G.borneensis* resembles that of *G.similis* and *G.corrugata*, but is weaker and more flattened than in those species. The spiral sculpture of *G.corrugata* is also more irregular. The adult shell *G.borneensis* is larger than in adult *G.similis* and *G.corrugata*. Additonally, the base to apex end sculpture of the protoconch of *G.borneensis* is distinct compared to these species (*G.similis* has a rounded protoconch sculpture and *G.corrugata* has straight-line protoconch sculpture).

######## Distribution.

The species is known only from Gomantong hill in the Kinabatangan region of Sabah.

######## Discussion.

The identification of *G.borneensis* can be confusing when we refer to the sketches by Thompson and Dance (1983: figs 61, 62). These appear to reflect the radial sculpture of *G.corrugata*, which is wavy/irregular and strongly sculpted. Smith (1895) and Thompson and Dance (1983) described *G.borneensis* referring to the absence of spiral sculpture, which, however, is present but only visible under high magnification. The bright red colour of the peristome as described by Smith (1895) could not be observed by us, probably due to the faded condition of the shells. We also find there is no association based on colouration of *G.borneensis* with *G.pyrrhoderma* and the “*williamsi*”. Thompson and Dance (1983) grouped the ‘*borneensis*’ based on their reddish shell colour, while ‘*williamsi*’ with their light brown colour, of which we find these colours are often a variation within these groups of species.

####### Georissa corrugata sp. n.

Taxon classificationAnimaliaCycloneritidaHydrocenidae

http://zoobank.org/81610B3D-0DB9-48ED-89D3-DAA69BDD031B

Figure 6A–I

######## Type locality.

Batu Tomanggong, Kinabatangan valley, Sandakan, Sabah, Malaysia (05°31.86'N, 118°18.24'E).

######## Type material.

**Figure 6.**
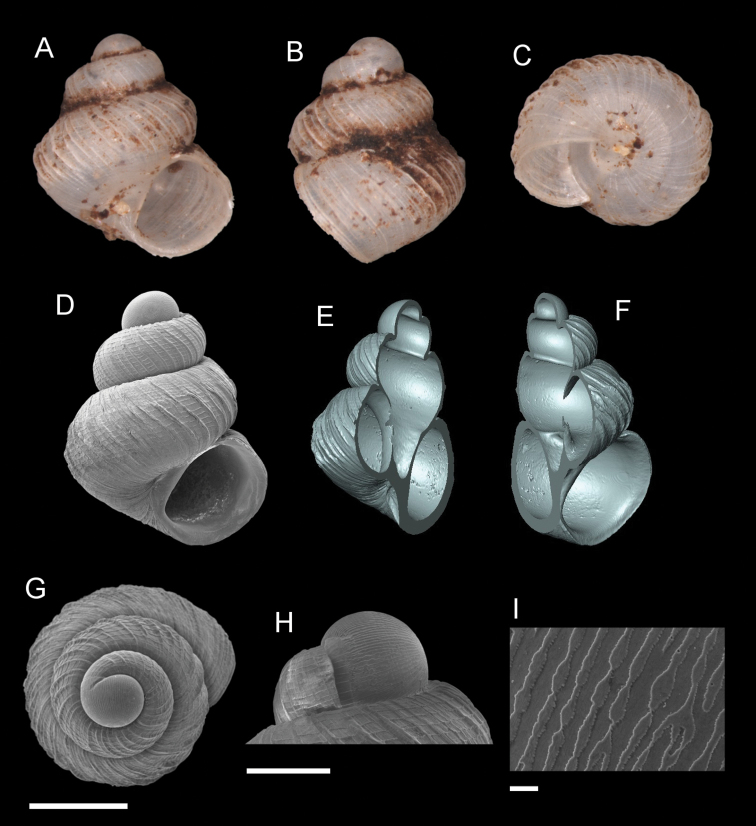
*Georissacorrugata* sp. n. **A–C** Holotype: MZU/MOL 16.15 **D–I** paratype: MZU/MOL 16.16 **A, D** shell apertural view **B** shell side view **C** shell rear view **E, F** shell cross-section from 3D model **G** shell top view **H** protoconch side view **I** close up of protoconch from top at 1000 × magnification. Scale bars: 500 µm (**A–G**); 200 µm (**H**); 10 µm (**I**).

*Holotype*. Batu Tomanggong, Kinabatangan valley, Sandakan, Sabah, Malaysia (05°31.86'N, 118°18.24'E): MZU/MOL 16.15 (Fig. 6A–C) *Paratypes*. Batu Tomanggong, Kinabatangan valley, Sandakan, Sabah, Malaysia (05°31.86'N, 118°18.24'E): MZU/MOL 16.16 (Fig. 6D–I). Batu Punggul, Sepulut valley, Sabah: JJV 1903 (1). N Borneo: RMNH/MOL 152848. Batu Keruak, Kinabatangan valley, Sabah (05°31.38'N, 118°17.10'E): BOR/MOL 1467, BOR/MOL 1844, BOR/MOL 11661 (1). Unnamed hill, Kinabatangan valley, Sabah (05°31.11'N, 118°17.23'E): BOR/MOL 2218 (1, juvenile).

######## Etymology.

The name is derived from a Latin word *corrugatus*, meaning “wrinkled”, referring to the coarse and irregular radial sculpture.

######## Description.

*Protoconch*. Colour: white. Sculpture pattern: parallel lines of varying width, widening before splitting in two. Mesh width: 3.1–6.2 µm, with the distance between each of the sculptural units as wide as the mesh width. *Teleoconch*. Colour: white. First whorl: rounded. Subsequent whorls: rounded. Suture: clearly impressed. Shoulder: narrow. Number of whorls: 2 ½–3. SH: 1.14–1.43 mm. SW: 1.01–1.11 mm. SI: 1.10–1.29. *Shell sculpture*. Radial sculpture: present, more prominent after the first whorl, wavy, irregular and widely spaced, often distinctly higher and strongly projected on and above the periphery. Spiral sculpture: present, regularly spaced, thin, discontinuous due the interruption by radial sculpture. *Aperture*. Shape: rounded to slightly ovoid, parietal side straight, palatal edge contiguous with the parietal side, palatal and basal sides convex. AH: 0.82–1.07 mm. AW: 1.00–1.09 mm. AI: 0.75–1.02. *Holotype dimensions*. SH: 1.71 mm, SW: 0.99 mm, AH: 0.52 mm, AW: 0.58 mm.

######## Diagnosis.

The wavy and irregular, widely spaced and strong radial sculpture, with thin regularly arranged spiral sculpture in between is diagnostic for *G.corrugata*. *Georissasimilis* and *G.borneensis* have a somewhat similar arrangement of radial and spiral sculpture, but do not carry the protoconch sculpture consisting of parallel lines. The shell shape and size of *G.corrugata* are similar to *G.similis*. Besides the difference in the protoconch sculpture, the latter species also has more densely arranged radial sculpture on the teleoconch.

######## Distribution.

*Georissacorrugata* is distributed on the limestone hills of the lower Kinabatangan valley, known to occur from Gomantong to Batu Tomanggong, but always in low densities compared to other *Georissa* species. The species has also been found in the Sepulut valley, ca. a hundred km further to the southwest.

####### Georissa everetti

Taxon classificationAnimaliaCycloneritidaHydrocenidae

Smith, 1895

Figures 1B , 7A–K

Georissa everetti Smith, 1895: 125, plate IV fig. 15; Thompson and Dance 1983: 120, figs 55–57.

######## Type locality.

Rumbang, W. Sarawak.

######## Type material.

*Holotype* (Holotype by original monotypy). Rumbang, Sarawak: NHMUK 1893.6.7.69 (glued on paper) (Fig. 1B) (Thompson and Dance 1983).

######## Other material.

**Figure 7.**
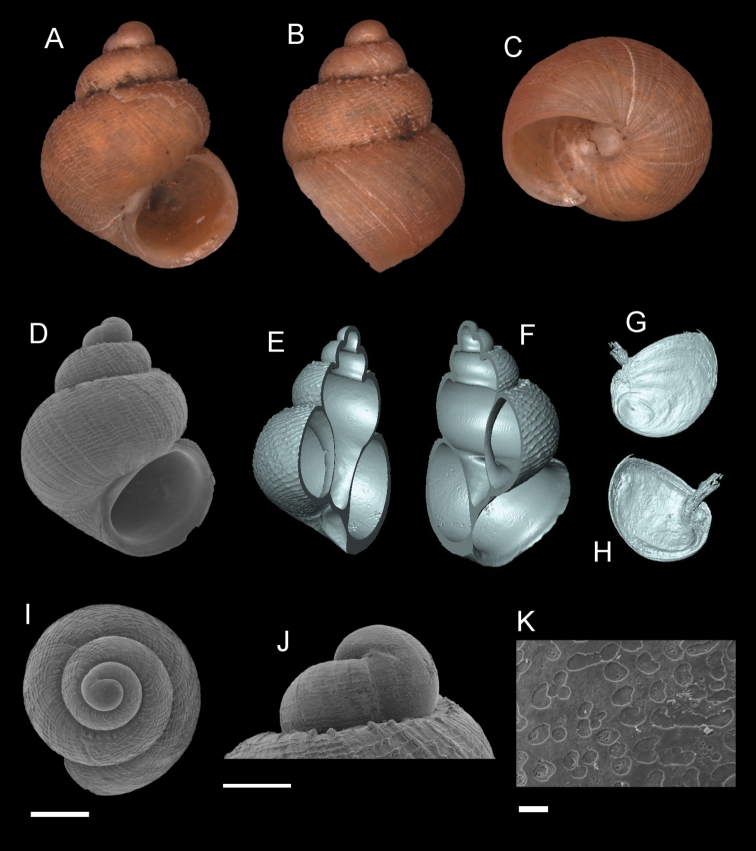
*Georissaeveretti* Smith, 1895. **A–K**JJV 10185 **A, D** shell apertural view **B** shell side view **C** shell rear view **E, F** shell cross-section from 3D model **G, H** operculum frontal and ventral view from 3D model **I** shell top view **J** protoconch side view **K** close up of protoconch from top at 1000 × magnification. Scale bars: 500 µm (**A–I**); 200 µm (**J**); 10 µm (**K**).

Kampung Giam, Lower Penrissen valley, Sarawak: JJV 12546. Kampung Benuk, Lower Penrissen valley, Sarawak (01°18.47'N, 110°17.29'E): JJV 12548. Kampung Temurang, Upper Penrissen valley, Sarawak (01°12.15'N, 110°16.18'E): JJV 12547. Great Cave, Niah National Park, Sarawak: RMNH/MOL 336264, JJV 10185 (Fig. 7), JJV 13119. Batu Punggul, Sepulut valley, Interior Province, Sabah: JJV 1906. Bukit Tinahas, Sepulut valley, Interior Province, Sabah (04°38.28'N, 116°37.05'E): JJV 7622.

######## Description.

*Protoconch*. Colour: orange to red. Sculpture: rounded to ellipsoidal mesh pattern, mixed with irregular sculptural shapes (whenever two or more rounded or ellipsoidal meshes are connected or combined). Mesh width: 4–30 µm. *Teleoconch*. Colour: orange to red. First whorl: convex. Subsequent whorls: convex, with relatively wide penultimate and final whorls. Suture: clearly impressed. Shoulder: narrow. Number of whorls: 2 ¾–3 ¼. SH: 1.82–2.23 mm. SW: 1.52–1.75 mm. SI: 1.16–1.30. *Shell sculpture*. Radial sculpture: present, thin, forming small nodules when intersecting with spiral sculpture; these nodules are also present on the shoulder close to the suture. Spiral sculpture: present, thin, regularly spaced, oblique, appearing immediately after the protoconch, distorted/discontinuous by radial ribs. *Aperture*. Shape: rounded to ovoid, straight to concave parietal side, palatal edge contiguous with the body whorl, basal side convex. AH: 1.05–0.92 mm. AW: 1.09–0.96 mm. AI: 0.89–0.99.

######## Diagnosis.

The strong and thin oblique spiral sculpture on its shell is diagnostic for *G.everetti*. *Georissasimilis* has a somewhat similar knitted sculpture pattern resulting from the intersection of radial and spiral ribbing, but the shell shape is entirely distinct, with broad penultimate and final whorls. Based on the shell shape and habitus, *G.everetti* resembles *G.gomantonensis* and *G.williamsi*, which, however, have clear, regular, spiral shell ribs.

######## Distribution.

*Georissaeveretti* is widely distributed in Sabah and Sarawak, but is found in low abundances. The species known to occur from Padawan/Penrissen, Sarawak in the South (where Rumbang, the type locality is located), to further north, Niah, Sarawak, and Sepulut valley, Sabah.

####### Georissa williamsi

Taxon classificationAnimaliaCycloneritidaHydrocenidae

Godwin-Austen, 1889

Figures 1C , 8A–I

Georissa williamsi Godwin-Austen, 1889: 353, Plate XXXIX fig. 10; Thompson and Dance 1983: 124 (**non**G.hungerfordi Godwin-Austen, 1889; G.javana Möllendorff, 1897; G.javanaintermedia Möllendorff, 1897).
Hydrocena williamsi (Godwin-Austen, 1889): Saul 1967: 109.
Georissa sp.1 (Godwin-Austen, 1889): Clements et al. 2008: Appendix D.

######## Type locality.

Borneo.

######## Type material.

*Holotype* (Holotype by original monotypy). Borneo: NHMUK 1889.12.7.71 (glued on paper) (Fig. 1C) (Thompson and Dance 1983).

######## Other material.

**Figure 8.**
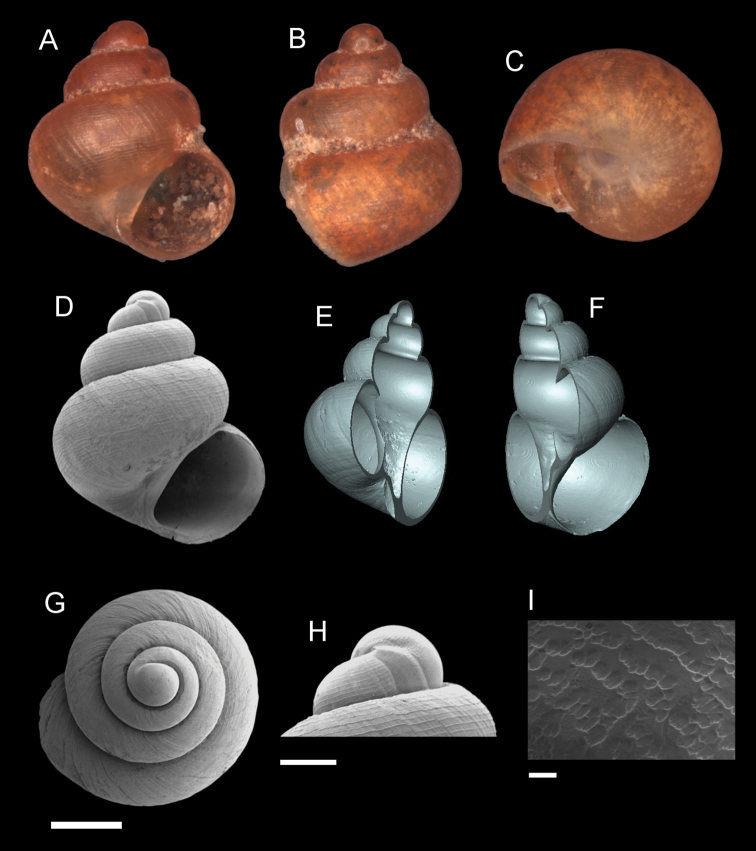
*Georissawilliamsi* Godwin-Austen, 1889. **A–I**RMNH/MOL 333946 **A, D** shell apertural view **B** shell side view **C** shell rear view **E, F** shell cross-section from 3D model **G** shell top view **H** protoconch side view **I** close up of protoconch from top at 1000 × magnification. Scale bars: 500 µm (**A–G**); 200 µm (**H**); 10 µm (**I**).

Batu Punggul, Sepulut valley, Interior province, Sabah (04°39.00'N, 116°37.00'E): RMNH/MOL 187642, BOR/MOL 57, JJV 1907. Gua Pungiton, Sepulut valley, Interior province, Sabah (04°42.41'N, 116°36.04'E): BOR/MOL 55, JJV 7543. Bukit Tinahas, Sepulut valley, East end of Batu Punggul limestone, Interior province, Sabah (04°38.28'N, 116°37.05'E): JJV 7623. Tinahas limestone hill, Interior Province, Sabah (04°38.46'N, 116°37.08'E): RMNH/MOL 333928, RMNH/MOL 334016, BOR/MOL 56, BOR/MOL 59. Batu Temurung, Sepulut valley, Interior province, Sabah (04°42.45'N, 116°34.40'E): BOR/MOL 58, BOR/MOL 60, JJV 8037. Simbaluyon limestone hill, Interior Province, Sabah, (04°43.25'N, 116°34.22'E): RMNH/MOL 333922, RMNH/MOL 333946 (Fig. 8), RMNH/MOL 334007. Batu Baturong ca. 50 km W.S.W. of Lahad Datu, Tawau province, Sabah (04°41.00'N, 118°1.00'E): JJV 1830. Madai limestone hill, Tawau Province, Sabah (04°43.66'N, 118°10.71'E): RMNH/MOL 337817, RMNH/MOL 337827, RMNH/MOL 337834. Cave on Teck Guan estate, Lahad Datu, Sabah: ZMA/MOLL 315607, ZMA/MOLL 315608, ZMA/MOLL 315609, ZMA/MOLL 315622.

######## Description.

*Protoconch*. Colour: orange to red. Sculpture: a mix of rounded, ellipsoidal to irregular sculptural shape. Mesh width: 2–6 µm. *Teleoconch*. Colour: orange to red. First whorl: convex. Subsequent whorls: convex. Suture: clearly impressed. Shoulder: narrow. Number of whorls: 3–3 ¼. SH: 1.58–1.91 mm. SW: 1.17–1.42 mm. SI: 1.30–1.38. *Shell sculpture*. Radial sculpture: absent, only weak to strong growth lines are visible at irregular intervals. Spiral sculpture: present, thin, regularly spaced at the first whorl, appearing immediately after the protoconch, on later whorls the spiral sculpture weakens and becomes distorted by the growth lines; more than 20 spiral ribs on the body whorl of the adult individual. *Aperture*. Shape: semi-elliptic, straight to concave parietal side, palatal side rounded, palatal edge contiguous with the body whorl, basal side slightly convex. AH: 0.63– 0.81 mm. AW: 0.71–0.87 mm. AI: 0.89–0.95.

######## Diagnosis.

*Georissawilliamsi* has a broad final whorl, in which it is similar to *G.gomantonensis* and *G.everetti*. However, these three species are all distinctly sculptured, where *G.gomantonensis* has raised spiral sculpture, *G.everetti* has oblique spiral sculpture, but *G.williamsi* has thin, hardly raised, and densely arranged spiral sculpture (4–6 ribs in every 0.1 mm), despite the similar shell habitus.

######## Distribution.

*Georissawilliamsi* occurs over a large part of Sabah from the Sepulut valley in the west-central to Tawau and Lahad Datu in the east.

######## Discussion.

The type locality of *G.williamsi* is ‘Borneo’, with no specific location stated by Godwin-Austen (1889). Saul (1966) in her note on “Shell collecting in the limestone cave of Borneo” mentioned that during her trip to Lahad Datu, Sabah, they collected *G.williamsi* (syn. *Hydrocenawilliamsi*). Based on the characters of *G.williamsi* described by Godwin-Austen (1889) and the type material we have examined, the species does not have very prominent spiral sculpture. *Georissawilliamsi* was previously misinterpreted as having highly raised spiral sculpture, and the name was therefore misapplied to forms like *G.hungerfordi*, *G.insulae*, and *G.javana* (Thompson and Dance 1983; Phung et al. 2017; Vermeulen and Whitten 1998).

####### Georissa trusmadi sp. n.

Taxon classificationAnimaliaCycloneritidaHydrocenidae

http://zoobank.org/B4441AFE-E2D5-4234-BE14-FDFDAEFAF3AE

Figures 9A–K

######## Type locality.

Loloposon Cave, Gunung Trus Madi, Sabah, Malaysia (05°39.00'N, 116°29.51'E).

######## Type material.

**Figure 9.**
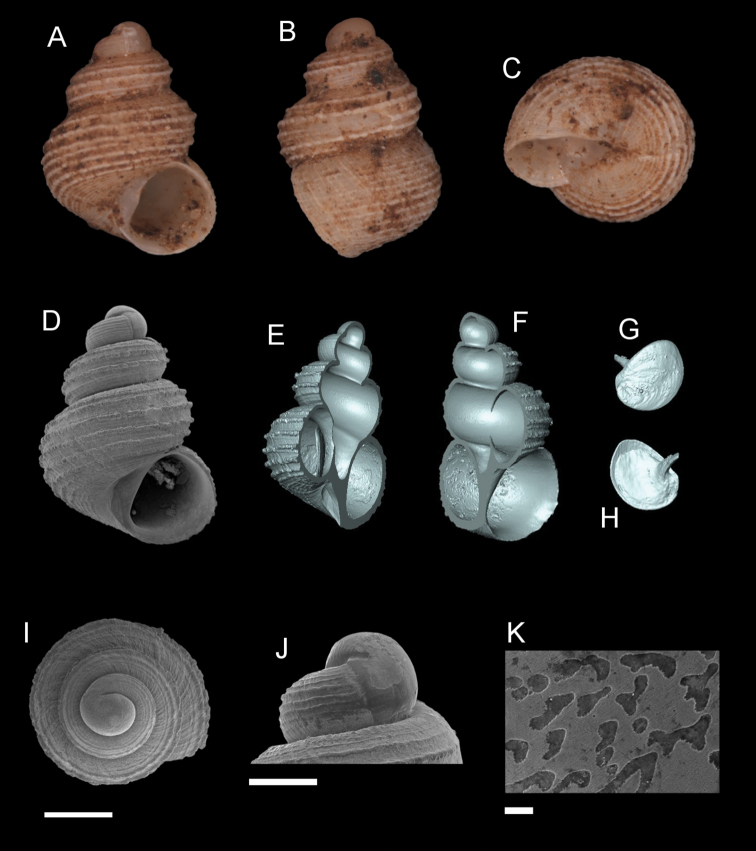
*Georissatrusmadi* sp. n. **A–C** Holotype: MZU/MOL 16.17 **D–K** paratype: MZU/MOL 16.18 **A, D** shell apertural view **B** shell side view **C** shell rear view **E, F** shell cross-section from 3D model **G, H** operculum frontal and ventral view from 3D model **I** shell top view **J** protoconch side view **K** close up of protoconch from top at 1000 × magnification. Scale bars: 500 µm (**A–I**); 200 µm (**J**); 10 µm (**K**).

*Holotype*. Loloposon Cave, Gunung Trus Madi, Sabah, Malaysia (05°39.00'N, 116°29.51'E): MZU/MOL 16.17 (Fig. 9A–C). *Paratypes*. Loloposon Cave, Gunung Trus Madi, Sabah, Malaysia (05°39.00'N, 116°29.51'E): MZU/MOL 16.18 (8) (Fig. 9D–K). Gunung Trus Madi slopes, Gua Loloposon, Interior province, Sabah (05°39.00'N, 116°29.51'E) (20): JJV 13231.

######## Etymology.

The species is named after the type locality, Gunung Trus Madi, Sabah.

######## Description.

*Protoconch*. Colour: orange. Sculpture pattern: rounded to irregular sculptural shape. Mesh width: 3–30 µm. *Teleoconch*. Colour: orange. First whorl: convex. Subsequent whorls: convex. Suture: well-impressed. Shoulder: narrow. Number of whorls: 2 ¾–3 ½. SH: 1.40–1.89 mm. SW: 1.12–1.37 mm. SI: 1.22–1.38. *Shell sculpture*. Radial sculpture: absent, weak growth lines present throughout the shell surface. Spiral sculpture: present, strong spiral ribs, broadly spaced above the whorls, ca. 5–7 strongly raised spiral ribs on the body whorl of the adult individual, appearing immediately after the protoconch, thin spiral ribs in between the stronger ones, more densely spaced and weaker at the basal part of the body whorl. *Aperture*. Shape: rounded to slightly ovoid, straight to convex parietal side, palatal edge contiguous with the parietal side, basal side convex. AH: 0.59– 0.72 mm. AW: 0.66–0.79 mm. AI: 0.85–0.91. *Holotype dimensions*. SH: 1.67 mm, SW: 1.28 mm, AH: 0.68 mm, AW: 0.75 mm.

######## Diagnosis.

*Georissatrusmadi* is characterised by the highly raised spiral sculpture. The number of strong spiral ribs on the first whorl is lower (3–5) than on the later whorls (5–7). The spiral sculpture is similar to *G.insulae* and *G.hungerfordi*, but always shows fewer ribs. Based on the shell habitus, it is similar to *G.hungerfordi* from Sarawak. The latter species, however, has the spiral ribs on the body whorl less strongly raised.

######## Distribution.

*Georissatrusmadi* is only known from Gunung Trus Madi, Sabah.

####### Georissa leucococca

Taxon classificationAnimaliaCycloneritidaHydrocenidae

Vermeulen, Liew & Schilthuizen, 2015

Figure 10A–I

Georissa leucococca Vermeulen et al., 2015: 33, fig. 19 A-B; Marzuki and Foon 2016: 317; Khalik et al. 2018: 2.

######## Type locality.

Malaysia, Sabah, Interior Province, Sepulut valley, Gua Pungiton (04°42.41'N, 116°36.04'E).

**Type material.***Holotype* (Holotype by original designation). Malaysia, Sabah, Interior Province, Sepulut valley, Gua Pungiton (04°42.41'N, 116°36.04'E): RMNH/MOL 5003956 (not seen, we were unable to locate the material in RMNH collection). *Paratypes*. Malaysia, Sabah, Interior Province, Sepulut valley, Gua Pungiton (04°42.41'N, 116°36.04'E): NHMUK 20150572, JJV 8081.

######## Other material.

**Figure 10.**
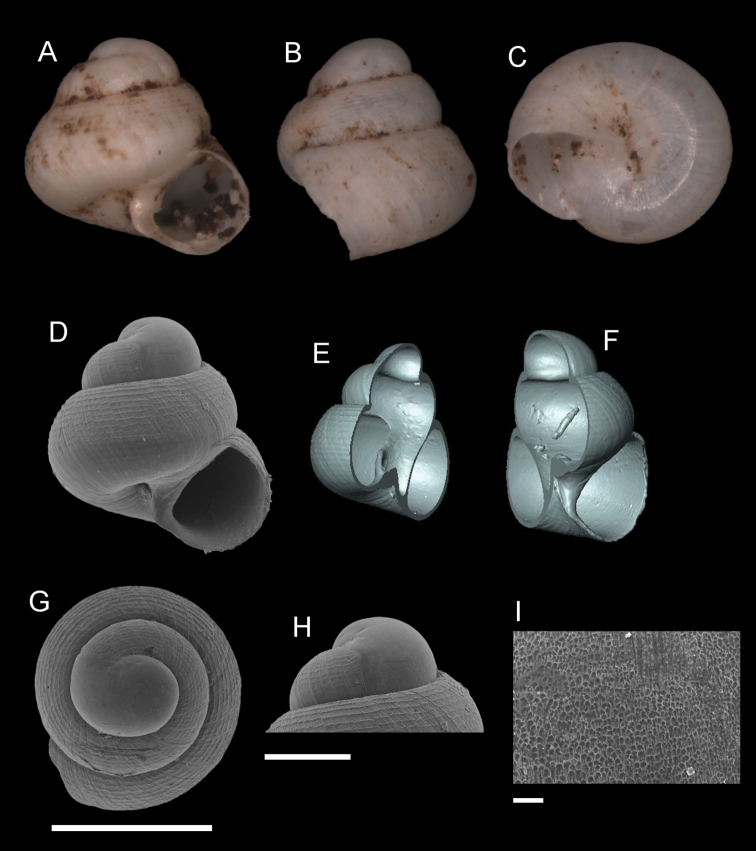
*Georissaleucococca* Vermeulen et al., 2015. **A–I**JJV 12572 **A, D** shell apertural view **B** shell side view **C** shell rear view **E, F** shell cross-section from 3D model **G** shell top view **H** protoconch side view **I** close up of protoconch from top at 1000 × magnification. Scale bars: 500 µm (**A–G**); 200 µm (**H**); 10 µm (**I**).

Gua Sanaron, Sepulut valley, Sabah (04°42.52'N, 116°36.16'E): JJV 8068. Gua Pungiton, Sepulut valley, Sabah: BOR/MOL 61. Gua Madai, Tawau province, Sabah (04°44.00'N, 118°8.00'E): JJV 1736. Batu Temurung, Sepulut valley, Sabah (04°42.45'N, 116°34.40'E): JJV 12681. Clearwater Cave, Mulu National Park, Sarawak: JJV 13098. Bukit Sarang group, Lower Tatau River valley, Sarawak: JJV 12571, JJV 12848, JJV 12849. Gunung Segu near Kampung Benuk, Penrissen valley, Sarawak (01°18.47'N, 110°17.29'E): JJV 12569. Bt. Krian, Upper Penrissen valley, Sarawak (01°12.20'N, 110°21.54'E): JJV 14217. Kampung Semedang, Lower Penrissen valley, Sarawak (01°17.49'N, 110°16.24'E): JJV 14221. Gunung Aup, Bau, Sarawak (01°21.36'N, 110°4.04'E): JJV 12570. Gunung Rapih, Bau, Sarawak (01°23.15'N, 110°8.29'E): JJV 12572 (Fig. 10). Gunung Chupak, Sungei Bukar headwaters, Sarawak (01°14.05'N, 110°20.50'E): JJV 14218, JJV 14219. Batu Staat, Sungei Sarawak Kiri valley, Sarawak (01°23.55'N, 110°14.55'E): JJV 14220.

######## Description.

*Protoconch*. Colour: white. Sculpture pattern: minutely formed, a mix of rounded, semi-elliptic to ellipsoidal. Mesh width: 1–2 µm. *Teleoconch*. Colour: white. First whorl: convex. Subsequent whorls: convex. Suture: well-impressed. Shoulder: narrow. Number of whorls: 2 ¼. SH: 0.62–0.72 mm. SW: 0.60–0.70 mm. SI: 0.97–1.06. *Shell sculpture*. Radial sculpture: absent, only weak growth lines at irregular intervals are visible. Spiral sculpture: present, thin, regularly spaced, appearing immediately after the protoconch, distorted by the growth lines, more prominent at the upper whorls, becoming weaker when closer to the columellar region. *Aperture*. Shape: semi-elliptic, straight to concave parietal side, palatal side rounded, palatal edge contiguous with the body whorl, basal side convex. Umbilicus: open, with a narrow space underneath the reflected columellar peristome. AH: 0.31– 0.37 mm. AW: 0.33–0.38 mm. AI: 0.87–0.97.

######## Diagnosis.

*Georissaleucococca* has spiral sculpture that is more prominent at the upper part of the whorls, similar to *G.bangueyensis*. *Georissaleucococca* is so far the only known Bornean *Georissa* with an open umbilicus and with an adult shell size of hardly more than 1 mm. It has an angular shell shape, similar to *G.borneensis*.

######## Distribution.

*Georissaleucococca* is widely distributed in Malaysian Borneo. The species is known to occur from west Sarawak to east Sabah.

####### Georissa hungerfordi

Taxon classificationAnimaliaCycloneritidaHydrocenidae

Godwin-Austen, 1889

Figures 1D , 11A–K

Georissa hungerfordi Godwin-Austen, 1889: 354, Plate XXXIX, fig. 9.
Georissa lowi Smith, 1893: 351.
Georissa williamsi Godwin-Austen: Thompson and Dance 1983: 124 (**non**G.williamsi Godwin-Austen, 1889; G.javana Möllendorff, 1897; G.javanaintermedia Möllendorff, 1897).

######## Type locality.

Borneo.

######## Type material.

*Lectotype* (Designation by Thompson and Dance 1983). Borneo: NHMUK 1891.3.17.864 (glued on paper) (Fig. 1D).

######## Other material.

**Figure 11.**
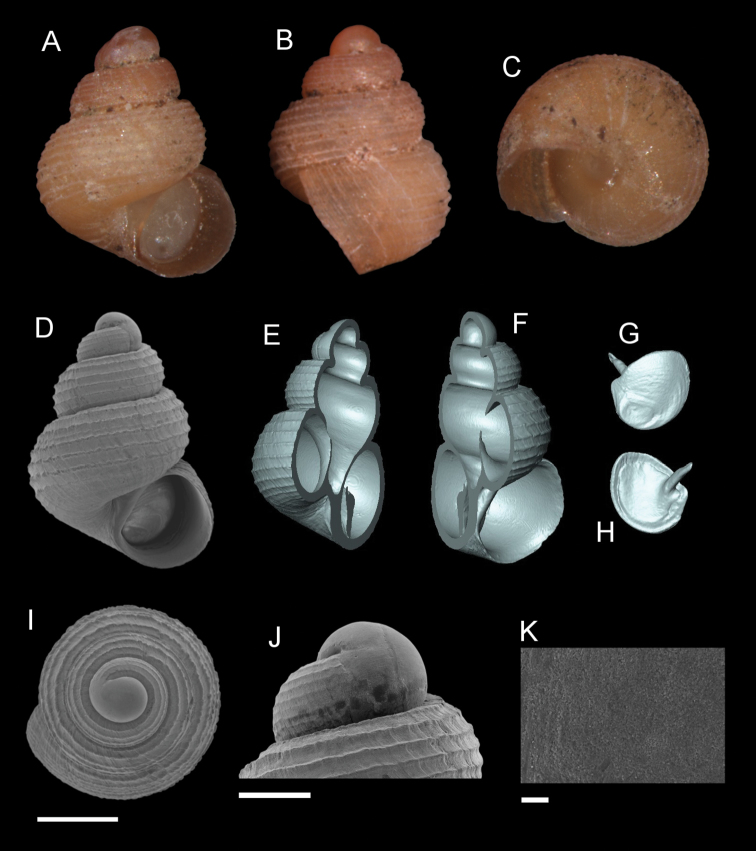
*Georissahungerfordi* Godwin-Austen, 1889. **A–K**MZU/MOL 16.11 **A, D** shell apertural view **B** shell side view **C** shell rear view **E, F** shell cross-section from 3D model **G, H** operculum frontal and ventral view from 3D model **I** shell top view **J** protoconch side view **K** close up of protoconch from top at 1000 × magnification. Scale bars: 500 µm (**A–I**); 200 µm (**J**); 10 µm (**K**).

Rumbang, Sarawak: NHMUK 1893.6.7.71, NHMUK 1893.6.7.108-110, NHMUK 94.7.21.58 (glued on paper), NHMUK 94.7.20.63-4 (glued on paper). Regu, Kampung Timurang, Padawan/Penrissen, Kuching, Sarawak (01°12.82'N, 110°16.82'E): MZU/MOL 16.10. Gunong Mawah, Kampung Bengoh, Padawan/Penrissan, Kuching, Sarawak (01°16.15'N, 110°15.46'E): MZU/MOL 16.11 (Fig. 11). Gunong Seduai/Duai, Kampung Timurang, Padawan/Penrissen, Kuching, Sarawak (01°12.25'N, 110°17.00'E): MZU/MOL 16.12. Gunong Sirat, Kampung Timurang, Padawan/Penrissen, Kuching, Sarawak (01°12.42'N, 110°16.52'E): MZU/MOL 16.13. Gunung Bra’ang, upper Penrissen valley, Kuching, Sarawak (01°14.12'N, 110°16.21'E): JJV 12451. Gunung Babu, upper Penrissen valley, Kuching, Sarawak (01°12.15'N, 110°16.18'E): JJV 12542. 12 km NNE of Padawan village, upper Penrissen valley, Kuching, Sarawak: JJV 13067, JJV 13070. Upper Penrissen valley, Bt. Krian, Kuching, Sarawak (01°12.20'N, 110°21.54'E): JJV 14222. Gunung Manok, upper Penrissen valley, Kuching, Sarawak (01°11.56'N, 110°16.16'E): JJV 14224. Gunung Kayan, upper Penrissen valley, Kuching, Sarawak (01°15.45'N, 110°15.30'E): JJV 14225. Sungei Bukar headwaters, G. Buros S of Gunung Nambi, Kuching, Sarawak (01°09.55'N, 110°27.59'E): JJV 14223. Gunung Pangga, Bau, Sarawak: JJV 2165. Gunung Jambusan, Bau, Sarawak: JJV 2213. Gunung Kapor, Bau, Sarawak: JJV 2274.

######## Description.

*Protoconch*. Colour: orange. Sculpture pattern: smooth. *Teleoconch*. Colour: orange. First whorl: convex. Subsequent whorls: convex, shell shape slender to broad. Suture: well-impressed suture, straight to slightly concave, and narrow shoulder. Number of whorls: 2 ½–3 ¼. SH: 1.35–1.85 mm. SW: 1.38–1.20 mm. SI: 1.16–1.36. *Shell sculpture*. Radial sculpture: absent, only weak to strong growth lines present throughout the shell surface. Spiral sculpture: present, strong spiral ribs, regularly spaced, with ca. 7–10 spiral ribs on the body whorl of the adult individual, appearing immediately after the protoconch, sometimes distorted/discontinuous by growth lines, more densely spaced spiral cords at the basal part of the body whorl. *Aperture*. Shape: rounded to slightly ovoid, straight to concave parietal side, palatal edge contiguous with the body whorl, basal side convex. AH: 0.63– 0.79 mm. AW: 0.67–0.83 mm. AI: 0.89–1.06.

######## Diagnosis.

*Georissahungerfordi* is characterised by the strong spiral sculpture with ca. 7–10 spiral ribs on the body whorl. The shell shape approaches the shape of *G.trusmadi* and *G.pachysoma*. *Georissahungerfordi* has stronger spiral sculpture compared to *G.pachysoma* but weaker compared with *G.trusmadi*. The spiral ribbings resemble *G.insulae*, which has, however, a more elongated and slender shell shape.

######## Distribution.

*Georissahungerfordi* is distributed from Bau to Padawan/Penrissen, Kuching, Sarawak.

######## Discussion.

Thompson and Dance (1983) considered *G.hungerfordi* a junior synonym of *G.williamsi*. We are, however, of the opinion that *G.hungerfordi* is a valid species based on the distinctly raised spiral sculpture of the shell compared to *G.williamsi*. *Georissalowi* (Smith, 1893) is a junior synonym of *G.hungerfordi*. See also discussion in *G.williamsi* Godwin-Austen, 1889.

######## Molecular analysis.

ML and Bayesian analyses show that the samples of *G.hungerfordi* (16S: n = 14; CO1: n = 11) form a monophyletic group with 100% BS and 100% PP, sister group to the “scaly” group, except *G.saulae*.

####### Georissa gomantonensis

Taxon classificationAnimaliaCycloneritidaHydrocenidae

Smith, 1893

Figures 1E , 12A–K

Georissa gomantonensis Smith, 1893: 351, Plate XXV fig. 25; Thompson and Dance 1983: 121, figs 36, 58–60; Schilthuizen et al. 2003: 41.
Georissa gomantongensis Smith: Vermeulen and Junau 2007: 217; Clements et al. 2008: 2762; Khalik et al. 2018: 19, fig. 1J.

######## Type locality.

Gomanton, N. Borneo.

######## Type material.

*Holotype* (Holotype by original monotypy). Gomanton, N. Borneo: NHMUK 1892.7.20.39 (glued on paper) (Fig. 1E) (Thompson and Dance 1983).

######## Other material.

**Figure 12.**
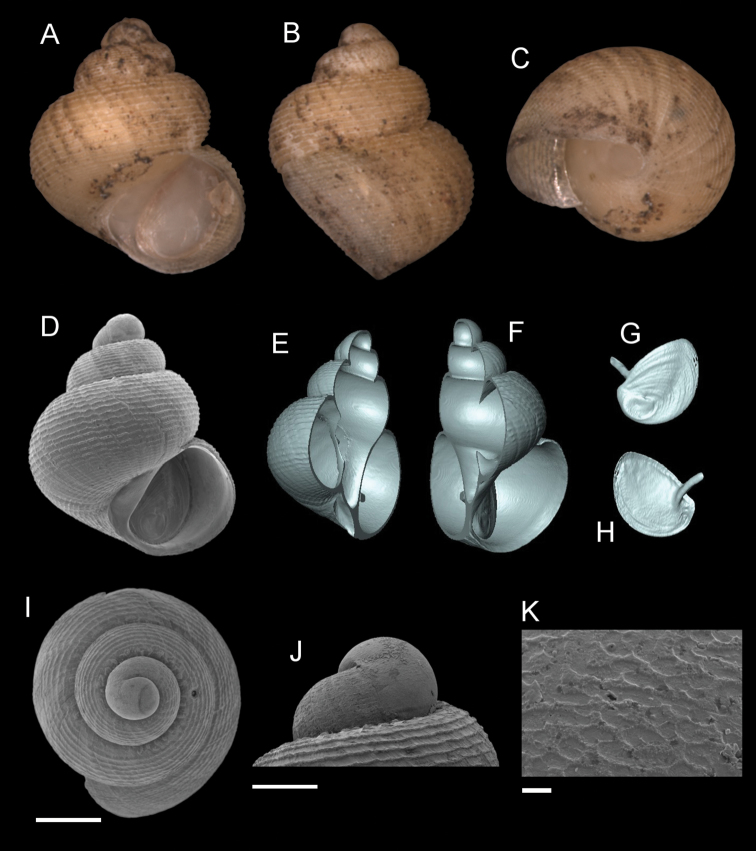
*Georissagomantonensis* Smith, 1893. **A–K** BOR/MOL 7389 **A, D** shell apertural view **B** shell side view **C** shell rear view **E, F** shell cross-section from 3D model **G, H** operculum frontal and ventral view from 3D model **I** shell top view **J** protoconch side view **K** close up of protoconch from top at 1000 × magnification. Scale bars: 500 µm (**A–I**); 200 µm (**J**); 10 µm (**K**). Shell view image (Fig. 12D) is the same image used in Khalik et al. (2018: fig. 1J).

Gua Gomantong, Kinabatangan, Sabah (05°32.00'N, 118°06.00'E): BOR/MOL 7632, BOR/MOL 7389 (Fig. 12), JJV 1612. Batu Tai (not Bod Tai) near Gomantong, Kinabatangan valley, Sabah (05°32.35'N, 118°10.32'E): JJV 9590. Batu Pangi, Kinabatangan valley, Sandakan province, Sabah (05°31.59'N, 118°18.43'E): BOR/MOL 10829, JJV 9648. Batu Keruak 2 near Sukau, Kinabatangan valley, Sabah (05°32.00'N, 118°18.00'E): JJV 9801. Kampung, Kinabatangan, Sabah (05°30.90'N, 118°16.86'E): BOR/MOL 10866, BOR/MOL 12545. Batu Tomanggong Besar 1, Kinabatangan, Sabah (05°31.26'N, 118°18.06'E): BOR/MOL 10561, BOR/MOL 11296. Batu Tomanggong Besar, lower Kinabatangan valley, Sandakan, Sabah (05°31.02'N, 118°18.21'E): BOR/MOL 2253, BOR/MOL 2282. Tomanggong 2, lower Kinabatangan valley, Sandakan, Sabah (05°31.00'N, 118°18.00'E): BOR/MOL 1462. Batu Keruak, Kinabatangan, Sabah (05°31.32'N, 118°17.10'E): BOR/MOL 1460, BOR/MOL 1883, BOR/MOL 11697. Bod Tai, Kinabatangan, Sabah (05°31.00'N, 118°13.00'E): BOR/MOL 1465, BOR/MOL 11256. Bukit Mawas, lower Kinabatangan valley, Sabah (05°27.00'N, 118°08.00'E): BOR/MOL 1463, BOR/MOL 1990. Unnamed hill 1, lower Kinabatangan valley, Sabah (05°31.11'N, 118°17.23'E): BOR/MOL 2152, BOR/MOL 2185. Unnamed hill 2, lower Kinabatangan valley, Sabah (05°30.00'N, 118°17.00'E): BOR/MOL 1461. Sabahmas Cave, Segama valley, Tawau, Sabah (05°08.52'N, 118°26.01'E): JJV 7452. Segama River, Segama valley, near bridge of road Sandakan to Lahad Datu, Tawau, Sabah (05°06.10'N, 118°13.12'E): JJV 7496. Tabin River, Segama valley, Sandakan, Sabah (05°18.49'N, 118°44.39'E): JJV 7753. Batu Temurung, Sepulut valley, Sabah (04°42.45'N, 116°34.40'E): JJV 8035. Pulau Mataking, Easternmost island of the Semporna-Sulu Chain, Sandakan: JJV 11523. Tabin Wildlife Reserve, Lahad Datu, Sabah (05°18.81'N, 118°44.65'E): BOR/MOL 19, BOR/MOL 20. Ulu Sungai Resang, lower Kinabatangan limestone hill, Sabah (05°31.00'N, 118°21.00'E): BOR/MOL 1464. N end of limestone ridge on E bank of Tabin River Sandakan Province, Sabah (05°18.04'N, 118°44.03'E): BOR/MOL 18. Batu Temurung, Sepulut Valley, Interior province, Sabah (04°42.04'N, 116°34.04'E): BOR/MOL 17.

######## Description.

*Protoconch*. Colour: greenish yellow. Sculpture: ellipsoidal mesh to irregular sculptural shape. Mesh width: 4–16 µm. *Teleoconch*. Colour: greenish yellow. First whorl: convex. Subsequent whorls: convex. Suture: well-impressed. Shoulder: slightly extended, with a row of regularly spaced granules. Number of whorls: 3–3 ¼. SH: 1.95–2.17 mm. SW: 1.67–1.68 mm. SI: 1.17–1.29. *Shell sculpture*. Radial sculpture: absent, only weak growth lines present throughout the shell surface. Spiral sculpture: present, strongly sculpted spiral ribs, at regular intervals, appearing immediately after the protoconch, sometimes distorted/discontinuous by growth lines, reduced in strength when reaching the columellar region, ca. 14–18 spiral ribs visible on the body whorl in the adult individual. *Aperture*. Shape: rounded to slightly ovoid, straight to concave parietal side, palatal edge contiguous with the body whorl, basal side convex. AH: 0.94– 0.97 mm. AW: 1.04–1.08 mm. AI: 0.87–0.92.

######## Diagnosis.

*Georissagomantonensis* is characterised by its bright greenish yellow colour, broad final whorl, and strong spiral sculpture. The spiral sculpture pattern is similar to *G.insulae*, but the shell habitus of *G.gomantonensis* is much broader and inflated. *Georissaeveretti* and *G.williamsi* have a similar shell habitus as *G.gomantonensis*, but differ because lacking of the oblique (*G.everetti*) and densely arranged (*G.williamsi*) spiral sculpture.

######## Distribution.

*Georissagomantonensis* is widely distributed throughout Sabah. More commonly found in the vegetation of the limestone forest, rather than on the limestone rocks themselves.

######## Molecular analysis.

ML and Bayesian analyses show that the individuals of *G.gomantonensis* (16S: n = 2; CO1: n = 2) form a monophyletic group with 100% BS and 100% PP, sister group to the paraphyletic *G.saulae* + *G.filiasaulae*.

####### Georissa filiasaulae

Taxon classificationAnimaliaCycloneritidaHydrocenidae

Haase & Schilthuizen, 2007

Figure 13A–K

Georissa filiasaulae Haase & Schilthuizen, 2007: 216, figs 2A–B and 2E; Clements et al. 2006: 736; Clements et al. 2008: Appendix D; Schilthuizen et al. 2012; Khalik et al. 2018.

######## Type locality.

Malaysia, Sabah, Sepulut valley, Interior province, Batu Sanaron (04°42.05'N, 116°36.01'E).

######## Type material.

*Holotype* (Holotype by original designation). Malaysia, Sabah, Sepulut valley, Interior province, Batu Sanaron (04°42.05'N, 116°36.01'E): BOR/MOL 3795. *Paratypes*. Malaysia, Sabah, Sepulut valley, Interior province, Batu Sanaron (04°42.05'N, 116°36.01'E): BOR/MOL 3491 (7); ZMB 107143-107149 (7) (not seen).

######## Other material.

**Figure 13.**
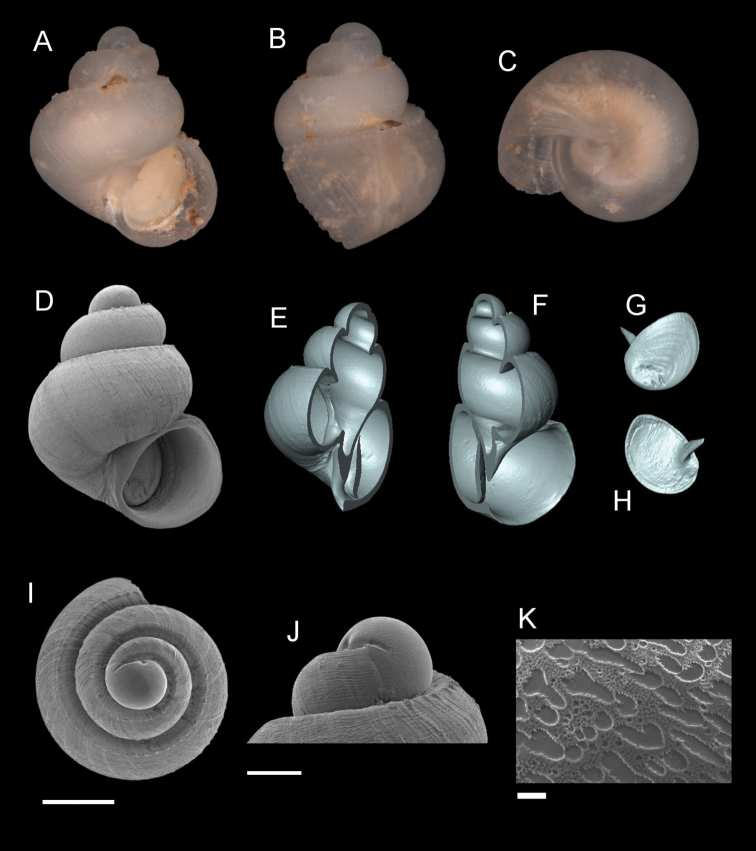
*Georissafiliasaulae* Haase & Schilthuizen, 2007. **A–K** BOR/MOL 12768 **A, D** shell apertural view **B** shell side view **C** shell rear view **E, F** shell cross-section from 3D model **G, H** operculum frontal and ventral view from 3D model **I** shell top view **J** protoconch side view **K** close up of protoconch from top at 1000 × magnification. Scale bars: 500 µm (**A–I**); 200 µm (**J**); 10 µm (**K**).

Batu Sanaron, Interior province, Sepulut valley, Sabah: BOR/MOL 532, BOR/MOL 3405. Batu Pungiton, Interior province, Sepulut valley, Sabah Batu Pungiton, Sabah (04°42.41'N, 116°36.04'E): BOR/MOL 12768 (Fig. 13).

######## Description.

*Protoconch*. Colour: white. Sculpture: a mix of rounded, ellipsoidal to irregular sculptural shape. Mesh width: 2.5–20 µm. *Teleoconch*. Colour: white. First whorl: convex. Subsequent whorls: convex, shell shape slender to broad. Suture: clearly impressed. Shoulder: slightly extended, regularly spaced nodules. Number of whorls: 2 ½–3. SH: 1.21–1.68 mm. SW: 1.67–1.68 mm. SI: 1.08–1.27. *Shell sculpture*. Radial sculpture: absent, weak to strong growth lines. Spiral sculpture: present, rather weak and thin, densely spaced on the first whorl, the ribbing appears immediately after the protoconch, sometimes distorted/discontinuous by the growth lines, superficially smooth on the later whorls. *Aperture*. Shape: rounded to semi-elliptic, straight to slightly convex parietal side, palatal side rounded, palatal edge partially contiguous with the body whorl and parietal side, basal side convex. AH: 0.67– 0.79 mm. AW: 0.69–0.83 mm. AI: 0.93–0.97.

######## Diagnosis.

*Georissafiliasaulae* has weak, thin, and densely arranged spiral sculpture with nodular structure on the shoulder. The shell colour and thickness are most similar to *G.corrugata*, which has white and partially transparent shell.

######## Distribution.

*Georissafiliasaulae* is a cave specialist, known from the cave system of Batu Sanaron and Batu Tinahas in the Sepulut valley. Schilthuizen et al. (2012) studied the population genetics of *G.filiasaulae* and its sister species, *G.saulae*. They found narrow hybrid zones between the two species in cave entrances.

######## Molecular analysis.

ML and Bayesian analyses of *G.filiasaulae* (16S: n = 3; CO1: n = 3) show that *G.filiasaulae* form one clade with 98% BS and 100% PP. The sister group is the *G.saulae* population from Pungiton (*G.saulae* is paraphyletic).

######## Discussion.

*Georissafiliasaulae* is one of the two known Bornean *Georissa* that is troglobitic. Khalik et al. (2018) described *G.silaburensis*, another species of Bornean *Georissa* from the “scaly” group as a possible troglobite from Gunung Silabur, Serian, Sarawak. *Georissafiliasaulae* differs from *G.saulae* by the absence of any scale-like sculpture, reduced shell pigmentation, and relatively larger shell size and broader shell shape. Population genetic studies suggest that the hybrid zone between the two is restricted to a narrow region at the cave entrances, rendering the two species as independent evolutionary units. Therefore, considering them as separate species is warranted (Schilthuizen 2000).

####### Georissa insulae sp. n.

Taxon classificationAnimaliaCycloneritidaHydrocenidae

http://zoobank.org/97E4E8EB-6926-4F20-9228-AB313291CD7B

Figure 14A–K

Georissa williamsi Godwin-Austen: Clements et al. 2008: Appendix D; Phung et al. (2017), fig. 8C.

######## Type locality.

Pulau Mantanani Besar, Sabah, Malaysia (06°43.06'N, 116°20.50'E).

######## Type material.

**Figure 14.**
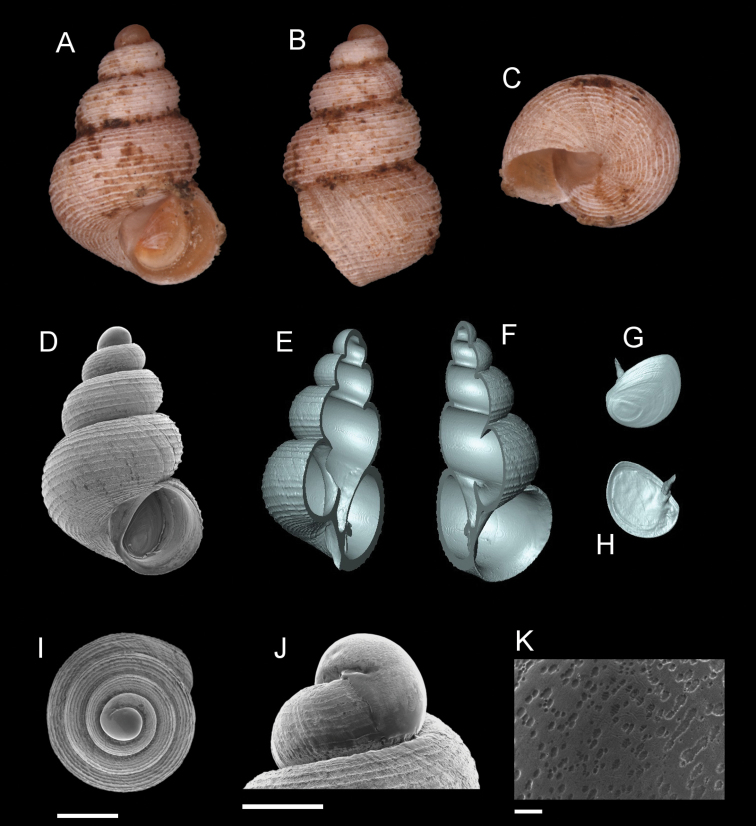
*Georissainsulae* sp. n. **A–C** Holotype: MZU/MOL 18.01 **D–K** paratype: MZU/MOL 18.02 **A, D** shell apertural view **B** shell side view **C** shell rear view **E, F** shell cross-section from 3D model **G, H** operculum frontal and ventral view from 3D model **I** shell top view **J** protoconch side view **K** close up of protoconch from top at 1000 × magnification. Scale bars: 500 µm (**A–I**); 200 µm (**J**); 10 µm (**K**).

*Holotype*. Pulau Mantanani Besar, Sabah, Malaysia (06°43.06'N, 116°20.50'E): MZU/MOL 18.01 (Fig. 14A–C). *Paratypes*: Pulau Mantanani Besar, Sabah, Malaysia (06°43.06'N, 116°20.50'E): MZU/MOL 18.02 (Fig. 14D–K). Pulau Mantanani Besar, West Coast Province, Sabah: JJV 9845 (8), JJV 9860 (>50), BOR/MOL 3718, BOR/MOL 7161 (1), BOR/MOL 7174 (9). Pulau Lungisan, Sabah: BOR/MOL 3744. Kinabalu N.P., Poring Hot Springs, along path to waterfall, West Coast Province, Sabah: JJV 13003 (1). Gua Mundau, Pitas, Sabah (06°33.02'N, 116°52.07'E): BOR/MOL 4373 (1, broken shell).

######## Etymology.

The name is a genitive singular of the Latin word *insula*, meaning ‘island’, which refers to the Mantanani islands, the main collecting locality.

######## Description.

*Protoconch*. Colour: orange. Sculpture pattern: a mix of rounded and ellipsoidal sculpture. Mesh width: 1.5–12.0 µm. *Teleoconch*. Colour: orange. First whorl: convex. Subsequent whorls: convex. Suture: well-impressed. Shoulder: narrow. Number of whorls: 2 ¾–3 ¼. SH: 1.78–2.11 mm. SW: 1.21–1.40 mm. SI: 1.42–1.51. *Shell sculpture*. Radial sculpture: absent, weak growth lines present throughout the shell surface. Spiral sculpture: present, strong, regularly spaced, ca. 10–12 spiral ribs on the body whorl of the adult individual, developed immediately after the protoconch; more densely spaced spiral ribs at the basal part of the body whorl, becoming weaker closer to the columellar region. *Aperture*. Shape: rounded to slightly ovoid, straight to concave parietal side, palatal edge contiguous with the body whorl, basal side convex. AH: 0.66–0.79 mm. AW: 0.74–0.84 mm. AI: 0.89–0.94. *Holotype dimensions*. SH: 2.11 mm, SW: 1.40 mm, AH: 0.79 mm, AW: 0.84 mm.

######## Diagnosis.

*Georissainsulae* is characterised by the strong and regularly spaced spiral ribs throughout the entire shell. This shell sculpture is similar to that of *G.hungerfordi* and *G.trusmadi*, but less raised than in these two species. *Georissainsulae*has a greater number of spiral ribs, ca. 10–12 ribs on its shell compared to these two species. The shell habitus is distinctly elongated compared to other spirally ribbed Bornean *Georissa*.

######## Distribution.

Known from islands of Mantanani Kecil, Mantanani Besar, Lungisan, and on the mainland from Pitas to Kinabalu National Park, Sabah.

######## Molecular analysis.

ML and Bayesian analyses of *G.insulae* (16S: n = 4) show that *G.filiasaulae* form one clade with 100% BS and 100% PP. Sister to the rest of “non-scaly” *Georissa*, except for *G.hungerfordi* + *G.gomantonensis* + *G.filiasaulae*.

####### Georissa pachysoma

Taxon classificationAnimaliaCycloneritidaHydrocenidae

Vermeulen & Junau, 2007

Figure 15A–K

Georissa pachysoma Vermeulen & Junau, 2007: 216, fig. 7.

######## Type locality.

Malaysia, Sarawak, 2^nd^ div.: Lower Tatau River valley, Bukit Sarang group, Bukit Lebik.

######## Type material.

*Holotype* (Holotype by original designation). Malaysia, Sarawak, 2^nd^ div.: Lower Tatau River valley, Bukit Sarang group, Bukit Lebik: RMNH/MOL 109084. *Paratypes*. Malaysia, Sarawak, 2^nd^ div.: Lower Tatau River valley, Bukit Sarang group, Bukit Lebik: JJV 12628 (10), JJV 12837 (2).

**Figure 15.**
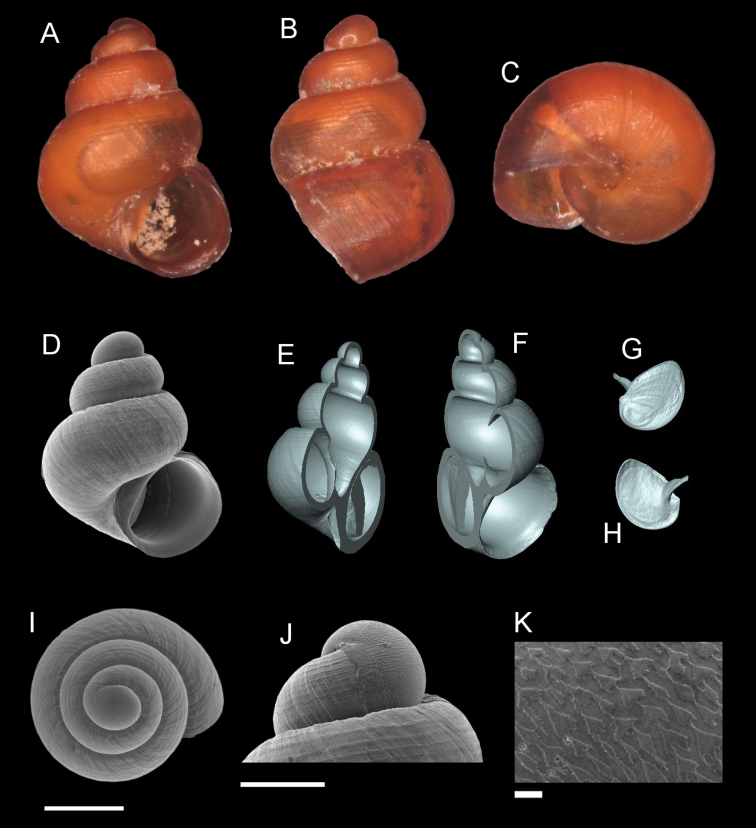
*Georissapachysoma* Vermeulen & Junau, 2007. **A–K**MZU/MOL 17.64 **A, D** shell apertural view **B** shell side view **C** shell rear view **E, F** shell cross-section from 3D model **G, H** operculum frontal and ventral view from 3D model **I** shell top view **J** protoconch side view **K** close up of protoconch from top at 1000 × magnification. Scale bars: 500 µm (**A–I**); 200 µm (**J**); 10 µm (**K**).

######## Other material.

Bukit Sarang group, Lower Tatau River valley: JJV 12626, JJV 12844, JJV 12845, JJV 12846. Upper Tatau River valley, upper Kakus River limestone scarps: JJV 12847. Bt. Besungai 0.5 m SW of Batu Gading, Long Lama, Baram valley (03°52.00'N, 114°25.00'E): JJV 4940. Slopes and cliffs along path to Great Cave, Niah National Park: JJV 10216. N side of limestone area, Painted Cave, Niah National Park: JJV 10391. Bukit Lebik and Bukit Anyi, Bukit Sarang, Bintulu, Sarawak (02°39.31'N, 113°02.47'E): MZU/MOL 17.62–MZU/MOL 17.84.

######## Description.

*Protoconch*. Colour: red to brown. Sculpture: a mix of triangular, rounded, and irregular sculptural shapes. Mesh width: 2–12 µm. *Teleoconch*. Colour: red to brown. First whorl: convex. Subsequent whorls: convex, broad at the final whorl. Suture: clearly impressed. Shoulder: narrow. Number of whorls: 2 ¾–3 ¼. SH: 1.20–1.65 mm. SW: 0.95–1.23 mm. SI: 1.19–1.34. *Shell sculpture*. Radial sculpture: absent, weak growth lines at irregular intervals only. Spiral sculpture: present, rather thin, widely spaced in the centre of the whorls, densely spaced close to the suture and the periphery, ribs appear immediately after the protoconch, ca. 12–15 medium raised spiral ribs, distorted/discontinuous where they are crossed by the growth lines. *Aperture*. Shape: rounded to semi-elliptic, straight to concave parietal side, palatal side convex and tilted below, palatal edge contiguous with the body whorl, basal side convex. AH: 0.54– 0.70 mm. AW: 0.57–0.72 mm. AI: 0.90–1.00.

######## Diagnosis.

*Georissapachysoma* is characterised by a dark red to brown colour of the shell, similar to *G.nephrostoma*, but the latter species has wavy spiral ribs while *G.pachysoma* does not. In shell habitus, *G.pachysoma* closely resembles *G.hungerfordi*, but the colour and spiral sculpture of *G.hungerfordi* (orange in colour in living or freshly dead specimens, with highly raised spiral sculpture) is entirely different from *G.pachysoma*.

######## Distribution.

*Georissapachysoma* is widely distributed from Bukit Sarang, Bintulu to further north in Baram and Niah, Sarawak.

######## Molecular analysis.

ML and Bayesian analyses of *G.pachysoma* (16S: n = 4; CO1: n = 4) show that *G.pachysoma* forms one clade with 100% BS and 100% PP, sister to the rest of the “non-scaly” group species, except for *G.hungerfordi* + *G.gomantonensis* + *G.filiasaulae* + *G.insulae*.

####### Georissa similis

Taxon classificationAnimaliaCycloneritidaHydrocenidae

Smith, 1893

Figures 1F , 16A–K

Georissa similis Smith, 1893: 351, Plate XXV fig. 26; Thompson and Dance 1983: 126, figs 37, 42, 73–75.
Georissa sp. 3 (Smith, 1893): Clements et al. 2008: Appendix D.

######## Type locality.

Gomanton Hill, N. Borneo.

######## Type material.

*Lectotype* (Designation by Thompson and Dance 1983). Gomanton Hill, N. Borneo: NHMUK 1892.7.23.51 (glued on paper) (Fig. 1F). *Paralectotype*. Gomanton Hill, N. Borneo: NHMUK 1892.7.23.52 (1) (glued on paper).

######## Other material.

**Figure 16.**
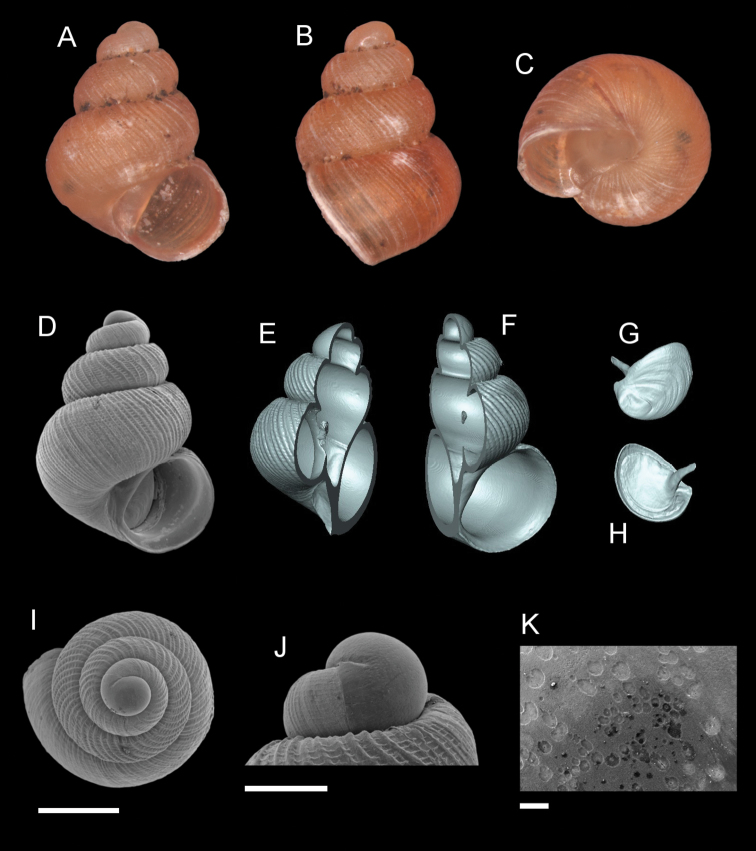
*Georissasimilis* Smith, 1893. **A–K**MZU/MOL 16.14 **A, D** shell apertural view **B** shell side view **C** shell rear view **E, F** shell cross-section from 3D model **G, H** operculum frontal and ventral view from 3D model **I** shell top view **J** protoconch side view **K** close up of protoconch from top at 1000 × magnification. Scale bars: 500 µm (**A–I**); 200 µm (**J**); 10 µm (**K**).

Gomanton, N. Borneo: NHMUK 94.7.20.59-60 (glued on paper), NHMUK 94.7.21.50-3 (glued on paper). Gomantong hill, Kinabatangan valley, Sandakan, Sabah (05°32.00'N, 118°06.00'E): JJV 1614. Gua Gomantong, Sabah (05°31.03'N, 118°04.01'E): BOR/MOL 52, BOR/MOL 3644. Bukit Mawas, lower Kinabatangan valley, Sabah (05°27.20'N, 118°08.67'E): BOR/MOL 1989. Batu Pangi, Kinabatangan valley, Sandakan, Sabah (05°31.59'N, 118°18.43'E): JJV 9831. Batu Tai (not Bod Tai) near Gomantong, Kinabatangan valley, Sandakan, Sabah (05°32.35'N, 118°10.32'E): JJV 9830, BOR/MOL 2686. Batu Keruak, lower Kinabatangan valley, Sabah (05°31.00'N, 118°17.00'E): BOR/MOL 1466. Ulu Sungai Resang, lower Kinabatangan, Sabah (05°31.00'N, 118°21.00'E): BOR/MOL 1447. Batu Batangan, Sabah (05°27.54'N, 118°06.18'E): MZU/MOL 16.14 (Fig. 16). Gua Madai, Tawau, Sabah (04°44.00'N, 118°08.00'E): BOR/MOL 53, JJV 1738, JJV 7693. Segarong Hills, Bukit Pababola, Semporna, Tawau, Sabah (04°33.00'N, 118°25.00'E): JJV 1772, JJV 1817. Batu Baturong, Tawau, Sabah (04°41.00'N, 118°01.00'E): JJV 1829, BOR/MOL 1446. Limestone hill on N bank Segama river, Tawau, Sabah (05°06.10'N, 118°13.12'E): JJV 7823. Tabin Wildlife reserve, Lahad Datu, Sabah (05°18.81'N, 118°44.65'E): BOR/MOL 54.

######## Description.

*Protoconch*. Colour: red. Sculpture: rounded to ellipsoidal mesh pattern. Mesh width: 2.8–7.0 µm. *Teleoconch*. Colour: red. First whorl: convex to flat at the upper part of the whorl. Subsequent whorls: convex. Suture: clearly impressed. Shoulder: narrow to slightly extended. Number of whorls: 2 ½–3 ¼. SH: 0.96–1.44 mm. SW: 0.85–1.06 mm. SI: 1.13–1.36. *Shell sculpture*. Radial sculpture: present, dense and regularly spaced, always stronger than the spiral sculpture. Spiral sculpture: present, raised but thin, appearing immediately after the protoconch, spiral sculpture often interrupted due to highly developed radial ribs; the overlapping radial and spiral sculptures form knitted structures on the shell. *Aperture*. Shape: semi-elliptic to rounded, straight to slightly concave parietal side, palatal edge contiguous with the parietal side, basal side convex. AH: 0.49– 0.64 mm. AW: 0.50–0.67 mm. AI: 0.85–0.98.

######## Diagnosis.

*Georissasimilis* is characterised by the dense radial sculpture. The radial ribs intersect with the thin spiral ribs and form knitted structures throughout the shell surface. The sculpture pattern is similar to *G.everetti* but not oblique, and the radial sculpture is more raised in *G.similis*. The shell shape is similar to *G.corrugata* and *G.xesta*, but these species differ entirely in their shell and protoconch sculpture (*G.corrugata* has irregular radial shell sculpture and straight-line protoconch sculpture; *G.xesta* does not have radial sculpture and the protoconch sculpture is a mix of irregular shapes).

######## Distribution.

*Georissasimilis* is widely distributed in the east of Sabah, from Sandakan in the north to Tawau in the south and Lahad Datu in the east.

######## Molecular analysis.

ML and Bayesian analyses of *G.similis* (16S: n = 5; CO1: n = 5) show that *G.similis* form one clade with 100% BS and 100% PP, sister to the group of *G.xesta* + *G.nephrostoma* + *G.bangueyensis* + *G.flavescens*.

######## Discussion.

Uribe et al. (2016) have published the mitochondrial genome of *G.similis* (GenBank acc. no. KU342664) which was previously identified as *G.bangueyensis* (see phylogenetic trees, Fig. 2A and B). Phylogenetic analyses have shown that it is possible to identify the identity of a *Georissa* even when shell data are not available.

####### Georissa xesta

Taxon classificationAnimaliaCycloneritidaHydrocenidae

Thompson & Dance, 1983

Figures 1G , 17A–K

Georissa xesta Thompson & Dance, 1983: 125, figs 69–70.

######## Type locality.

A small limestone ridge quarried for rock 5 mi W Kudat, Sabah, Borneo (06°57.00'N, 116°48.00'E).

######## Type material.

*Holotype* (Holotype by original designation). A small limestone ridge quarried for rock 5 mi W Kudat, Sabah, Borneo (06°57.00'N, 116°48.00'E): UF 35968 (not seen). *Paratypes*. A small limestone ridge quarried for rock 5 mi W Kudat, Sabah, Borneo (06°57.00'N, 116°48.00'E): UF 35969 (not seen), UF35970 (not seen), SMF 255740/6 (not seen), NHMUK 1984.006 (5) (Fig. 1G), JJV 13424.

######## Other material.

**Figure 17.**
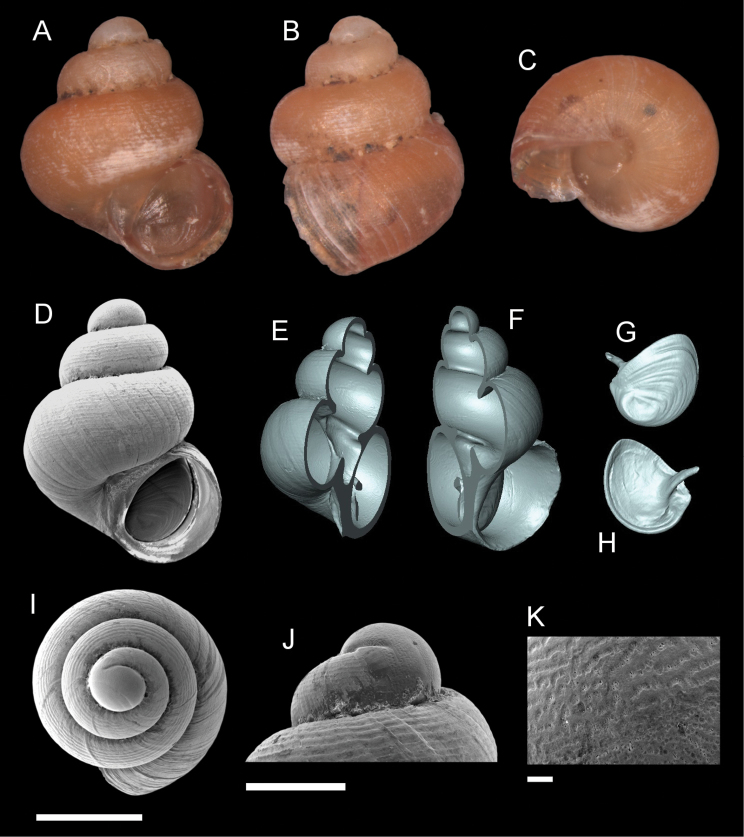
*Georissaxesta* Thompson & Dance, 1983. **A–K** BOR/MOL 7303 **A, D** shell apertural view **B** shell side view **C** shell rear view **E, F** shell cross-section from 3D model **G, H** operculum frontal and ventral view from 3D model **I** shell top view **J** protoconch side view **K** close up of protoconch from top at 1000 × magnification. Scale bars: 500 µm (**A–I**); 200 µm (**J**); 10 µm (**K**).

Kinabatangan valley, Batu Tulug (Batu Putih) along road Lahad Datu-Sandakan, N of bridge over Kinabatangan River, Sandakan province, Sabah (05°25.00'N, 117°56.00'E): JJV 1481. Kinabatangan valley, Batu Keruak 2 near Sukau, Sandakan province, Sabah (05°32.00'N, 118°18.00'E): JJV 9786. Kinabatangan valley, Batu Tomanggong Kecil, Sandakan province, Sabah (05°30.12'N, 118°18.10'E): JJV 9828. Batu Tomanggong Besar, Kinabatangan valley, Sandakan, Sabah (05°31.02'N, 118°18.21'E): BOR/MOL 1437, BOR/MOL 2252, BOR/MOL 2281. Batu Tomanggong Besar 2, Kinabatangan valley, Sandakan, Sabah (05°31.16'N, 118°18.33'E): BOR/MOL 1440. Batu Tomanggong Kecil, Kinabatangan valley, Sandakan, Sabah (05°30.21'N, 118°18.18'E): BOR/MOL 2025, BOR/MOL 2053. Batu Keruak, Kinabatangan valley, Sandakan, Sabah (05°32.00'N, 118°18.00'E): BOR/MOL 2687. Lower Kinabatangan valley, Sabah; Unnamed limestone hill 1 (05°31.11'N, 118°17.23'E): BOR/MOL 2151, BOR/MOL 2184, BOR/MOL 2217; Unnamed limestone hill 2 (05°30.00'N, 118°17.00'E): BOR/MOL 1441. Batu Materis, Kinabatangan valley, Sabah (05°31.21'N, 118°01.31'E): BOR/MOL 2113, BOR/MOL 2083. Bod Tai, Kinabatangan valley, Sabah (05°31.00'N, 118°13.00'E): BOR/MOL 1443. Bukit Mawas, lower Kinabatangan valley, Sabah: BOR/MOL 1444. Pangi, Kinabatangan valley, Sandakan province, Sabah (05°31.59'N, 118°18.43'E): BOR/MOL 1442. Ulu Sungai Resang, lower Kinabatangan valley, Sabah (05°31.00'N, 118°21.00'E): BOR/MOL 1438, BOR/MOL 7303 (Fig. 17), BOR/MOL 7311. Segama valley, ‘Kirk’s Cave’ 8 km N of Lahad Datu, Tawau province, Sabah (05°04.00'N, 118°16.00'E): JJV 1236. Segama valley, hill NW of crossing road Sandakan-Lahad Datu with the Segama River, Tawau province, Sabah (05°06.00'N, 118°13.00'E): JJV 1687. Segama valley, Sabahmas Cave, Tawau province, Sabah (05°08.52'N, 118 26.01'E): JJV 7453. Batu Baturong, N slope, Tawau province, Sabah (04°41.46'N, 118°0.45'E): JJV 7583. Segama valley, N end of limestone ridge on East bank Tabin River, Sandakan province, Sabah (05°18.49'N, 118°44.39'E): JJV 7755. Tabin Wildlife Reserve, Lahad Datu, Sabah: BOR/MOL 12, BOR/MOL 13. Limestone hill on N bank Segama River, Tawau province, Sabah (05°06.01'N, 118°13.01'E): BOR/MOL 9. Sabahmas Cave, Tawau Province, Sabah (05°08.05'N, 118°26.00'E): BOR/MOL 8. Batu Baturong, Tawau Province, Sabah (04°41.04'N, 118°00.04'E): BOR/MOL 10. N end of limestone ridge on E bank Tabin River, Sandakan Province, Sabah (05°18.04'N, 118°44.03'E): BOR/MOL 11. Tomanggong Sukau, Sandakan, Sabah (05°32.01'N, 118°23.00'E): BOR/MOL 14. Sabah, Malaysia: RMNH/MOL 335369. Sabah, N. Borneo: ZMA/MOLL 315545. Materis, Kinabatangan, Sabah (05°31.38'N, 118°01.02'E): BOR/MOL 7258.

######## Description.

*Protoconch*. Colour: pale orange to orange. Sculpture: irregular sculptural shape resulted from a combination of rounded to ellipsoidal sculpture patterns. Mesh width: 1–10. *Teleoconch*. Colour: orange, the colour of the teleoconch always darker than the protoconch. First whorl: convex. Subsequent whorls: convex. Suture: clearly impressed. Shoulder: narrow to slightly extended. Number of whorls: 2 ¾–3 ½. SH: 1.05–1.68 mm. SW: 0.84–1.18 mm. SI: 1.22–1.45. *Shell sculpture*. Radial sculpture: absent, only weak growth lines present. Spiral sculpture: present, ca. 20–25 thin and weak spiral ribs, superficially smooth, densely arranged, appearing immediately after the protoconch, distorted by growth lines. *Aperture*. Shape: rounded to slightly ovoid, straight to concave parietal side, palatal edge partially contiguous with the body whorl and the parietal side, basal side convex. AH: 0.48– 0.66 mm. AW: 0.50–0.74 mm. AI: 0.76–0.98.

######## Diagnosis.

*Georissaxesta* has densely arranged spiral sculpture (8–10 ribs in every 0.1 mm), unlike *G.bangueyensis* (4–5 ribs in every 0.1 mm), which has more space in between the spiral ribs. The shell of *G.xesta* looks superficially smooth under a stereomicroscope at low contrast with less than × 20 magnification. The dense spiral sculpture is similar to the spiral ribbing pattern of *G.williamsi*, but the shell habitus of these two species is entirely different, where *G.williamsi* has a broad ultimate whorl but *G.xesta* does not. Based on the shell shape, *G.xesta* is similar to *G.similis* and *G.corrugata*, but both of these species have strongly raised radial sculpture.

######## Distribution.

*Georissaxesta* is widely distributed in Sabah, especially in the coastal areas around Kudat, Sandakan, Lahad Datu, and Tawau.

######## Molecular analysis.

In the ML and Bayesian analyses of *G.xesta* (16S: n = 2; CO1: n = 3), the Materis and Ulu Resang populations form highly supported clades 96% BS and 100% PP, which are paraphyletic with respect to *G.nephrostoma*.

######## Discussion.

The type series of *G.xesta* from NHMUK seems to be partially eroded. However, the densely arranged thin spiral sculpture which is the diagnostic character of the species is still visible in the type series.

####### Georissa nephrostoma

Taxon classificationAnimaliaCycloneritidaHydrocenidae

Vermeulen, Liew & Schilthuizen, 2015

Figure 18A–K

Georissa nephrostoma Vermeulen et al., 2015: 34, fig. 20.

######## Type locality.

Malaysia, Sabah, Sandakan Province, Kinabatangan valley, Batu Keruak 2 near Sukau.

######## Type material.

*Holotype* (Holotype by original designation). Malaysia, Sabah, Sandakan Province, Kinabatangan valley, Batu Keruak 2 near Sukau (05°32.00'N, 118°18.00'E): RMNH/MOL 5003955 (not seen, we were unable to locate the material in RMNH collection). *Paratypes*. Malaysia, Sabah, Sandakan Province. Kinabatangan valley, Batu Keruak 2 near Sukau (05°32.00'N, 118°18.00'E): NHMUK 20150573, BOR/MOL 1449, BOR/MOL 1450, BOR/MOL 1845, JJV 9795; Batu Pangi (05°31.59'N, 118°18.43'E): BOR/MOL 1452, JJV 9833; Batu Tai near Gomantong (05°32.35'N, 118°10.32'E): JJV 9832; Tandu Batu (05°35.47'N, 118°20.34'E): JJV 9834; Limestone hills near Sukau Police Station: BOR/MOL 2186, BOR/MOL 2153, BOR/MOL 1451.

######## Other material.

**Figure 18.**
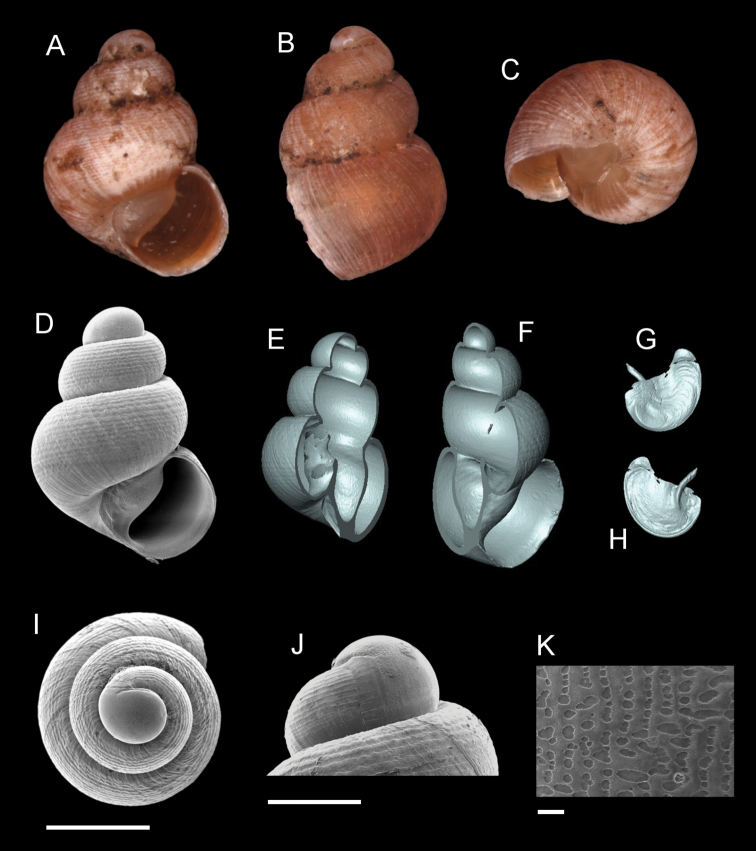
*Georissanephrostoma* Vermeulen et al., 2015. **A–K**MZU/MOL 17.29 **A, D** shell apertural view **B** shell side view **C** shell rear view **E, F** shell cross-section from 3D model **G, H** operculum frontal and ventral view from 3D model **I** shell top view **J** protoconch side view **K** close up of protoconch from top at 1000 × magnification. Scale bars: 500 µm (**A–I**); 200 µm (**J**); 10 µm (**K**). Shell image of the apertural view (Fig. 18D) is the same as shown in Khalik et al. (2018: fig. 1K).

Batu Keruak, Sandakan Province, Sabah (05°32.00'N, 118°18.00'E): BOR/MOL 1454, MZU/MOL 17.29 (Fig. 18). Tandu Batu, Sandakan Province, Sabah (05°35.47'N, 118°20.34'E): BOR/MOL 2685.

######## Description.

*Protoconch*. Colour: red to brown. Sculpture: rounded, ellipsoidal to irregular sculptural shape. Mesh width: 2–10 µm. *Teleoconch*. Colour: red to brown. First whorl: rounded to convex. Subsequent whorls: rounded to convex. Suture: clearly impressed. Shoulder: narrow. Number of whorls: 2 ½–2 ¾. SH: 0.87–1.24 mm. SW: 0.69–0.92 mm. SI: 1.26–1.43. *Shell sculpture*. Radial sculpture: absent, densely spaced weak to strong growth lines, no formation of true radial ribs. Spiral sculpture: present, appearing immediately after the protoconch; the ribs are low but narrow to broadly sculpted, regularly spaced, wavy, ca. 12–14 spiral ribs at the upper part of the body whorl; near the aperture, the spiral sculpture is weakened and flattened approaching the columellar region. *Aperture*. Shape: semi-elliptic, highly convex, and bulky parietal side, palatal side rounded, palatal edge contiguous with the parietal side, basal side convex. AH: 0.40–0.55 mm. AW: 0.43–0.60 mm. AI: 0.92–0.95.

######## Diagnosis.

*Georissanephrostoma* is characterised by the wavy formation of the spiral sculpture and the inflated parietal side of the aperture. The wavy sculpture pattern of *G.nephrostoma* is similar to *G.flavescens* but the two species differ entirely based on the shell habitus, where *G.flavescens* has a more compressed shell habitus. In shell shape, *G.nephrostoma* resembles *G.similis*, *G.xesta* and *G.bangueyensis*, but none of these species have the uniquely inflated parietal side of the aperture.

######## Distribution.

*Georissanephrostoma* is distributed from Sukau to Gomantong, Kinabatangan region, Sabah.

######## Molecular analysis.

ML and Bayesian analyses of *G.nephrostoma* (16S: n = 5; CO1: n = 1) showed that all *G.nephrostoma* specimens form one clade with 99% BS and 100% PP. The sister group is the *G.xesta* population from Materis and Ulu Resang (*G.xesta* is paraphyletic).

####### Georissa bangueyensis

Taxon classificationAnimaliaCycloneritidaHydrocenidae

Smith, 1895

Figures 1H , 19A–K

Georissa bangueyensis Smith, 1895: 125, Plate IV fig. 16; Thompson and Dance 1983: 126.

######## Type locality.

Banguey Island, N. Borneo.

######## Type material.

*Lectotype* (Designation by Thompson and Dance 1983). Banguey Island, N. Borneo: NHMUK 1893.6.7.9 (Fig. 1H).

######## Other material.

**Figure 19.**
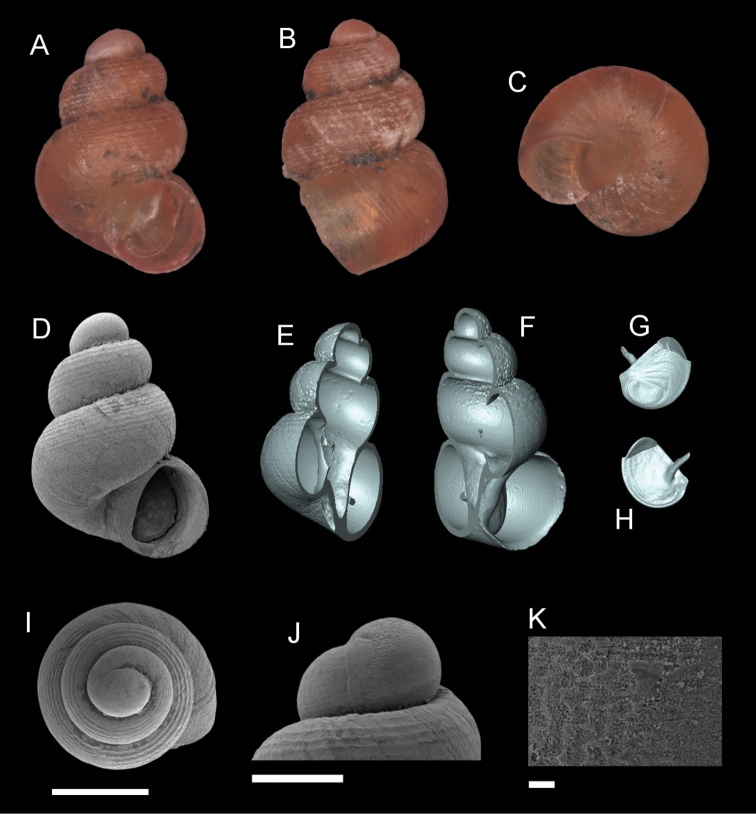
*Georissabangueyensis* Smith, 1895. **A–K**RMNH/MOL 5004968 **A, D** shell apertural view **B** shell side view **C** shell rear view **E, F** shell cross-section from 3D model **G, H** operculum frontal and ventral view from 3D model **I** shell top view **J** protoconch side view **K** close up of protoconch from top at 1000 × magnification. Scale bars: 500 µm (**A–I**); 200 µm (**J**); 10 µm (**K**).

Banggi Island, South end, Kudat province, Sabah (07°06.32'N, 117°5.07'E): RMNH/MOL 152746, JJV 1423, JJV 1451, JJV 9467, JJV 9497. Pulau Banggi, Kudat Dist., Sabah: BOR/MOL 15. Bod Gaya Island, Tun Sakaran Marine Park, Semporna, Sabah: BOR/MOL 4729. Gomantong limestone hill, Sabah: BOR/MOL 3320. Balambangan Island, Sabah (07°14.00'N, 116°52.00'E): BOR/MOL 3684. Kok simpul, Pulau Balambangan, Kudat Province, Sabah (07°13.03'N, 116°53.14'E): BOR/MOL 1445. S end Batu Sireh, Pulau Balambangan, Kudat Province, Sabah (07°12.29'N, 116°51.30'E): BOR/MOL 1439. Segama valley, limestone hill on N bank Segama River, near bridge of road Sandakan to Lahad Datu, Tawau province, Sabah (05°06.10'N, 118°13.12'E): JJV 7495. Materis, Kinabatangan, Sabah (05°31.38'N, 118°01.02'E): BOR/MOL 11820, BOR/MOL 11851, BOR/MOL 11910, BOR/MOL 11945. Bukit Mawas, lower Kinabatangan valley, Sabah (05°27.20'N, 118°08.67'E): BOR/MOL 1954, RMNH/MOL 5004968 (Fig. 19). Kampung, Kinabatangan, Sabah (05°30.72'N, 118°16.92'E): BOR/MOL 10901, BOR/MOL 10922. Ulu Resang, Kinabatangan, Sabah (05°30.66'N, 118°20.40'E): BOR/MOL 9284, BOR/MOL 9311, BOR/MOL 9345, BOR/MOL 9595, BOR/MOL 9610, BOR/MOL 9617. Batu Payung, Kinabatangan, Sabah (05°35.34'N, 118°19.44'E): BOR/MOL 8952, BOR/MOL 8967, BOR/MOL 8976, BOR/MOL 9003. Tomanggong Kecil, Kinabatangan, Sabah (05°30.54'N, 118°17.94'E): RMNH/MOL 152858, RMNH/MOL 152859, BOR/MOL 7473, BOR/MOL 9619, BOR/MOL 9685, BOR/MOL 9903, BOR/MOL 9943, BOR/MOL 9952, BOR/MOL 9983. Tomanggong Besar 1, Kinabatangan, Sabah (05°31.86'N, 118°18.24'E): BOR/MOL 10560, BOR/MOL 10806, BOR/MOL 11318, BOR/MOL 11361. Tomanggong Besar 2, Kinabatangan, Sabah (05°31.32'N, 118°17.88'E): BOR/MOL 10385, BOR/MOL 10411, BOR/MOL 10531, BOR/MOL 11352. “NewLocation1”, Kinabatangan, Sabah (05°27.40'N, 118°08.76'E): RMNH/MOL 5004826.

######## Description.

*Protoconch*. Colour: red. Sculpture: irregular sculptural shape to smooth. Mesh width: 1–20 µm. *Teleoconch*. Colour: red. First whorl: convex. Subsequent whorls: convex. Suture: clearly impressed. Shoulder: narrow. Number of whorls: 2 ¾–3. SH: 1.00–1.33 mm. SW: 0.77–0.96 mm. SI: 1.22–1.42. *Shell sculpture*. Radial sculpture: absent, only weak growth lines are here and there visible. Spiral sculpture: present, appearing immediately after the protoconch, regularly arranged; ca. 8–10 spiral ribs on the first whorl, on the later whorls the sculpture is more prominent at the upper part of the whorl, weaker and flattened closer to the columellar region. *Aperture*. Shape: semi-elliptic to rounded, straight to slightly concave parietal side, palatal edge contiguous with the parietal side, basal side convex. AH: 0.40– 0.53 mm. AW: 0.45–0.60 mm. AI: 0.81–1.00.

######## Diagnosis.

*Georissabangueyensis* is characterised by its clear spiral ribs at the upper part of the body whorl, similar to *G.flavescens* and *G.nephrostoma*, but the two latter species have wavy spiral ribs. Spiral sculpture on the lower whorl is weaker and less obvious closer to the columellar region. In shell sculpture, it is most similar to *G.xesta*, but the latter species has more densely arranged spiral sculpture (see discussion in *G.xesta*).

######## Distribution.

*Georissabangueyensis* is widely distributed in the coastal regions of northern and eastern Sabah.

######## Molecular analysis.

ML and Bayesian analyses of *G.bangueyensis* (16S: n = 6; CO1: n = 6) show that *G.bangueyensis* forms a monophyletic clade with 100% BS and 100% PP, and is sister to *G.flavescens*.

######## Discussion.

Thompson and Dance (1983) questioned the validity *G.bangueyensis* as a proper species based on a limited number of specimens. We propose that *G.bangueyensis* is a proper species with distinct characteristics, as compared to *G.xesta*.

####### Georissa flavescens

Taxon classificationAnimaliaCycloneritidaHydrocenidae

Smith, 1895

Figures 1I , 20A–K

Georissa flavescens Smith, 1895: 126, Plate IV fig. 17; Thompson and Dance 1983: 121.

######## Type locality.

Gomanton, N.E. Borneo.

######## Type material.

*Lectotype* (Designation by Thompson and Dance 1983). Gomanton, NE Borneo: NHMUK 1893.6.8.11 (Fig. 1I). *Paralectotypes*. Gomanton, NE Borneo: NHMUK 1893.6.8.12-13.

######## Other material.

**Figure 20.**
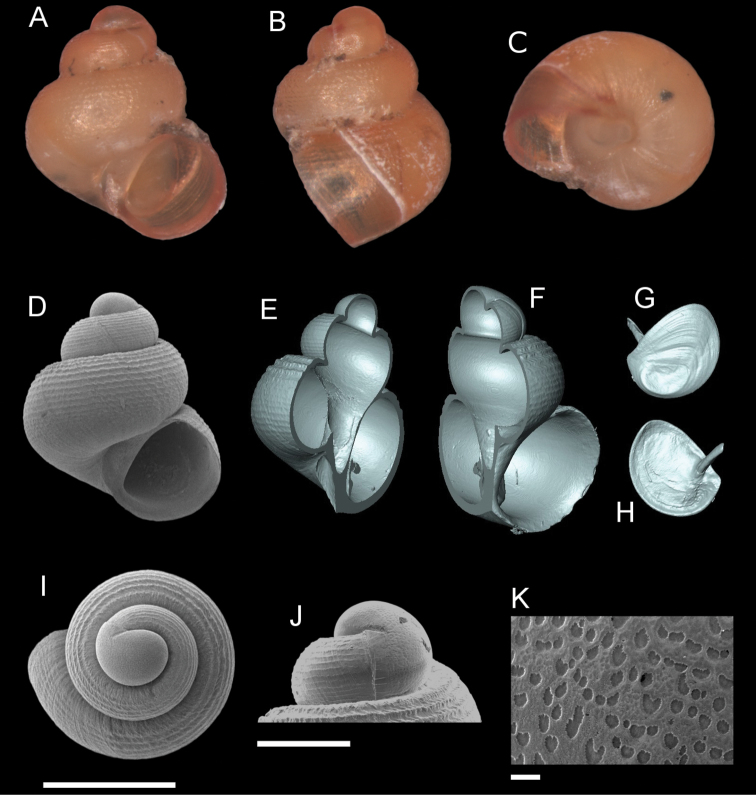
*Georissaflavescens* Smith, 1895. **A–K** BOR/MOL 7288 **A, D** shell apertural view **B** shell side view **C** shell rear view **E, F** shell cross-section from 3D model **G, H** operculum frontal and ventral view from 3D model **I** shell top view **J** protoconch side view **K** close up of protoconch from top at 1000 × magnification. Scale bars: 500 µm (**A–I**); 200 µm (**J**); 10 µm (**K**).

Tomanggong Besar 1, Kinabatangan, Sabah: BOR/MOL 7299. Tomanggong Besar 2, Kinabatangan, Sabah: BOR/MOL 7626. Batu Pangi, Kinabatangan valley, Sandakan province, Sabah (05°31.59'N, 118°18.43'E): JJV 9827, BOR/MOL 7288 (Fig. 20), BOR/MOL 9261, BOR/MOL 9325 (*G.flavescens* mixed with *G.xesta*), BOR/MOL 10816, BOR/MOL 10830, BOR/MOL 10835. Batu Keruak, Kinabatangan, Sabah (05°31.38'N, 118°17.16'E): BOR/MOL 11621.

######## Description.

*Protoconch*. Colour: orange. Sculpture: rounded, ellipsoidal to irregular sculptural shape. Mesh width: 2–10 µm. *Teleoconch*. Colour: orange. First whorl: convex. Subsequent whorls: convex, and slightly angular at the penultimate whorl. Suture: clearly impressed. Shoulder: narrow to slightly extended. Number of whorls: 2 ½–2 ¾. SH: 0.87–1.20 mm. SW: 0.73–0.95 mm. SI: 1.15–1.26. *Shell sculpture*. Radial sculpture: absent, weak growth lines. Spiral sculpture: present, appearing immediately after the protoconch, wavy, thin, and regularly arranged ribs at the first whorl, more raised at the later whorls. *Aperture*. Shape: semi-elliptic, straight to concave parietal side, palatal side rounded, palatal edge contiguous with the palatal side, basal side convex. AH: 0.43– 0.55 mm. AW: 0.47–0.58 mm. AI: 0.82–0.95.

######## Diagnosis.

*Georissaflavescens* is characterised by the wavy spiral sculpture, which it only shares with *G.nephrostoma*, but the latter species, with its narrow spire and inflated columella, is entirely distinct in shell habitus. The shell shape of *G.flavescens* is similar to *G.gomantonensis*, *G.williamsi*, and *G.everetti*, but its size is reduced compared to these three species.

######## Distribution.

*Georissaflavescens* is restricted to four limestone hills, Batu Pangi, Batu Keruak, Batu Gomantong, and Batu Tomanggong, in the Lower Kinabatangan valley of Sabah.

######## Molecular analysis.

ML and Bayesian analyses of *G.flavescens* (16S: n = 7; CO1: n = 8) show that *G.flavescens* forms a monophyletic clade with 100% BS and 100% PP, a sister species of *G.bangueyensis*.

######## Discussion.

Thompson and Dance (1983) synonymised *G.flavescens* to *G.gomantonensis*, without stating any reason. We find otherwise, that *G.flavescens* is a valid species based on detailed conchology and molecular analysis.

## Supplementary Material

XML Treatment for Georissa borneensis

XML Treatment for Georissa corrugata

XML Treatment for Georissa everetti

XML Treatment for Georissa williamsi

XML Treatment for Georissa trusmadi

XML Treatment for Georissa leucococca

XML Treatment for Georissa hungerfordi

XML Treatment for Georissa gomantonensis

XML Treatment for Georissa filiasaulae

XML Treatment for Georissa insulae

XML Treatment for Georissa pachysoma

XML Treatment for Georissa similis

XML Treatment for Georissa xesta

XML Treatment for Georissa nephrostoma

XML Treatment for Georissa bangueyensis

XML Treatment for Georissa flavescens

## Supplementary materials

Supplementary material 1 An overview of scanning parameters of each "non-scaly" *Georissa* Data type: table This dataset is made available under the Open Database License (http://opendatacommons.org/licenses/odbl/1.0/). The Open Database License (ODbL) is a license agreement intended to allow users to freely share, modify, and use this Dataset while maintaining this same freedom for others, provided that the original source and author(s) are credited.Mohd Zacaery Khalik, Kasper P. Hendriks, Jaap J. Vermeulen, Menno Schilthuizen

Supplementary material 2 Shell measurement of the "non-scaly" *Georissa* Data type: measurement data This dataset is made available under the Open Database License (http://opendatacommons.org/licenses/odbl/1.0/). The Open Database License (ODbL) is a license agreement intended to allow users to freely share, modify, and use this Dataset while maintaining this same freedom for others, provided that the original source and author(s) are credited.Mohd Zacaery Khalik, Kasper P. Hendriks, Jaap J. Vermeulen, Menno Schilthuizen
