# Supplementary material for: Conchological and molecular analysis of the “non-scaly” Bornean Georissa with descriptions of three new species (Gastropoda, Neritimorpha, Hydrocenidae)
Source: Zookeys. 2019 Apr 17;840:35–86. doi: 10.3897/zookeys.840.33326 (PMC6482118; doi:10.3897/zookeys.840.33326)
Supplement: Supplementary material 1 [file zookeys-840-035-s001.docx]

**Supplementary material 1**

Table S1.1 An overview of the scanning parameters of each examined “non-scaly” *Georissa* taxa.

| No. | Species | Filter type | Detector distance (mm) | Pixel size (µm) | Transmission  (%) | Exposure time (sec.) | Intensity |
| --- | --- | --- | --- | --- | --- | --- | --- |
| 1 | *G. bangueyensis* | LE1 | 60 | 1.6874 | 60-85 | 4.0 | 6300-8000 |
| 2 | *G. nephrostoma* | LE1 | 66 | 1.0380 | 65-90 | 13.0 | 5000-6500 |
| 3 | *G. similis* | LE1 | 60 | 1.6876 | 57-88 | 4.0 | 5700-8500 |
| 4 | *G. xesta* | LE1 | 50 | 1.3940 | 45-85 | 5.5 | 5000-9000 |
| 5 | *G. williamsi* | LE1 | 35 | 2.2927 | 40-80 | 9.0 | 5000-12000 |
| 6 | *G. borneensis* | LE2 | 31 | 2.4797 | 40-80 | 5.0 | 5000-13000 |
| 7 | *G. corrugata* | LE1 | 50 | 1.7132 | 45-80 | 7.0 | 5000-10000 |
| 8 | *G. insulae* | LE1 | 35 | 2.2929 | 50-80 | 10.0 | 5000-12000 |
| 9 | *G. trusmadi* | LE1 | 38 | 2.0864 | 40-80 | 9.0 | 5000-10000 |
| 10 | *G. pachysoma* | LE1 | 32 | 2.2502 | 35-75 | 9.0 | 5000-11000 |
| 11 | *G. flavescens* | LE1 | 73 | 0.9980 | 50-85 | 9.0 | 5000-10000 |
| 12 | *G. everetti* | LE2/LE5 | 25 | 3.6800 | - | 2.0 | 5100-7700 |
| 13 | *G. gomantongensis* | LE1/LE4 | 30 | 2.8561 | 45-87 | 1.0 | 6100-8250 |
| 14 | *G. leucococca* | LE2/LE5 | 60 | 1.3494 | - | 4.0 | 5900-7700 |
| 15 | *G. hungerfordi* | LE1 | 40 | 2.2500 | 6-80 | 1.0 | 15000-16000 |
| 16 | *G. filiasaulae* | LE2 | 30 | 3.0666 | 46-76 | 1.0 | 6000-9000 |
